# Serine-rich repeat proteins from gut microbes

**DOI:** 10.1080/19490976.2019.1602428

**Published:** 2019-04-29

**Authors:** Dimitrios Latousakis, Donald A. MacKenzie, Andrea Telatin, Nathalie Juge

**Affiliations:** The Gut Microbes and Health Institute Strategic Programme, Quadram Institute Bioscience, Norwich Research Park, Norwich, UK

**Keywords:** Serine rich repeat protein, protein glycosylation, adhesin, gut symbiont, Lactobacillus

## Abstract

Serine-rich repeat proteins (SRRPs) have emerged as an important group of cell surface adhesins found in a growing number of Gram-positive bacteria. Studies focused on SRRPs from streptococci and staphylococci demonstrated that these proteins are *O*-glycosylated on serine or threonine residues and exported via an accessory secretion (aSec) system. In pathogens, these adhesins contribute to disease pathogenesis and represent therapeutic targets. Recently, the non-canonical aSec system has been identified in the genomes of gut microbes and characterization of their associated SRRPs is beginning to unfold, showing their role in mediating attachment and biofilm formation. Here we provide an update of the occurrence, structure, and function of SRRPs across bacteria, with emphasis on the molecular and biochemical properties of SRRPs from gut symbionts, particularly Lactobacilli. These emerging studies underscore the range of ligands recognized by these adhesins and the importance of SRRP glycosylation in the interaction of gut microbes with the host.

## Introduction

**Figure 1.**
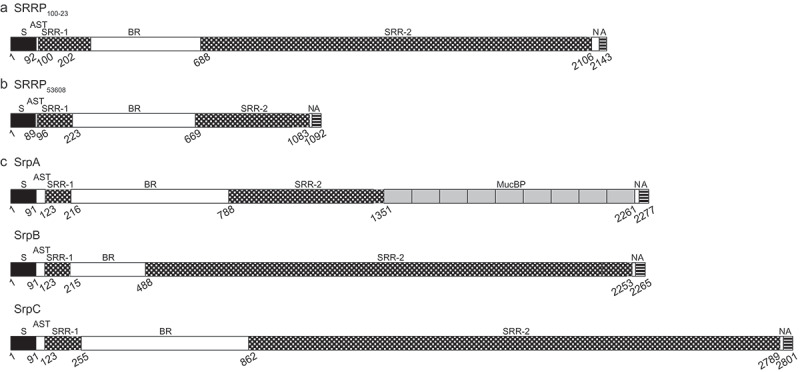
Structural domain organization of characterized serine-rich repeat proteins (SRRPs) from gut commensal bacteria. (a) *L. reuteri* strain 100–23, (b) *L. reuteri* strain ATCC 53608, (c) *Strep. salivarius* strain JIM8777. SRRPs are composed of an N-terminal signal peptide (S; black boxes); an AST domain; a non-repeat domain that mediates adhesion (binding region; BR); two serine-rich repeat domains (SRR) flanking the BR (SRR-1 and SRR-2; checkered boxes); and a second non-repeat domain (N), followed by a C-terminal cell wall anchor domain (A; striped boxes). In contrast to the majority of SRRPs, SrpA from *Strep. salivarius* strains, including JIM8777, also harbors a number of MucBP (mucin-binding protein) domains before and/or after SRR-2 (nine after SRR-2 in the case of JIM8777; grey boxes). The SRRs are composed of serine residues alternating with, most frequently, either an alanine, valine, or threonine residue. Numbers represent the starting amino acid positions of each domain. White boxes represent non-repeat regions.

SRRPs cover a functionally and structurally diverse family of glycoproteins found in many Gram-positive bacteria^1^. These proteins were originally identified in oral bacteria, such as streptococci and later in staphylococci,^2–11^ where their expression has been linked to virulence.^11–13^ Recently, SRRPs have also been reported in gut commensal microbes^14-16^ including *Lactobacillus reuteri*, a Gram-positive bacterial species inhabiting the gastrointestinal (GI) tract of vertebrates^17,18^ and *Streptococcus salivarius*, a pioneer colonizer and commensal bacterium of the human GI tract.^14^ SRRPs are composed of distinct subdomains: a cleavable and unusually long signal peptide which is followed by an accessory Sec transport (AST) domain, a short serine-rich repeat region (SRR-1), a binding region (BR) (also known originally as a ‘basic region’ due to its unusual composition of basic amino acids), a second and much larger SRR-2, and a short non-repeat region that includes a LPTXG cell wall anchoring motif^1,19,20^ (Figure 1). SRRP-BRs encompass a range of 3D structures, highlighting a relationship between their structural folds and binding ligands.

Export of SRRPs onto the bacterial surface occurs through a dedicated non-canonical Sec translocase, SecY2A2.^21^ This accessory secretion (aSec) system is encoded by genes that are normally co-located with the *srrp* gene(s) within a gene cluster and is composed of the motor protein SecA2, the translocon channel SecY2 and three to five accessory Sec proteins (Asp1-5). In addition, this gene cluster also contains genes encoding a variable number of glycosyltransferases (GTs), ranging between two to ten.^22^

**Figure 2.**
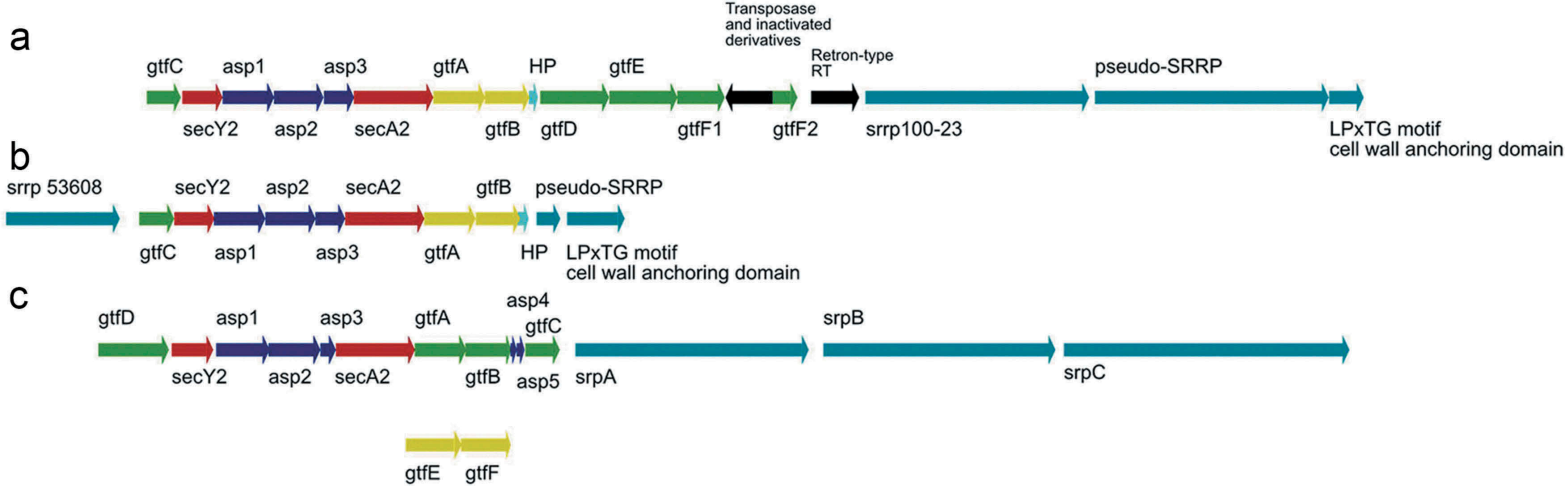
Organization of functionally characterized *secA2/Y2* clusters from gut commensal bacteria. (a) *L. reuteri* 100–23, (b) *L. reuteri* ATCC 53608 and (c) *Strep. salivarius* JIM8777. The genes encoding the SecA2/Y2 translocation machinery are shown in red, the accessory secretion proteins Asp1-5 in blue and the priming GTs, GtfA, and GtfB in *L. reuteri* and GtfE and GtfF in *Strep. salivarius*, in yellow. Genes encoding additional GTs are shown in green and the genes encoding SRRPs are illustrated in teal. Black arrows represent genes that are not part of the aSec machinery. HP: hypothetical protein.

Recent studies identified SecA2/Y2 clusters in the genomes of various *Lactobacillus* species,^16,18,23^ suggesting a conserved role of SRRPs among gut symbionts that possess the SecA2/Y2 cluster (Figure 2). One of the characteristics of SRRPs is that they undergo glycosylation, resulting in a range of glycan structures, reflecting the unique glycosylation pathways of the bacterial strains. It is expected that the combination of fold and glycosylation pattern will dictate the specificity of SRRPs in mediating the interaction of the bacteria with their respective host or niche within a host.

## Occurrence of SRRPs in bacterial genomes

A detailed bioinformatics analysis of all genome-sequenced bacteria classed as “non-pathogenic” or commensal revealed genes encoding full-length SRRPs and SecA2/Y2 secretion systems in a number of species from the order Lactobacillales, including strains of *L. reuteri, Lactobacillus oris, Lactobacillus salivarius, Lactobacillus johnsonii, Lactobacillus fructivorans, Lactococcus lactis, Streptococcus salivarius, Streptococcus vestibularis, Streptococcus mitis, Streptococcus oralis, Streptococcus cristatus*, and *Streptococcus* sp. strain DD12, with none found so far in other major lactobacilli species such as *Lactobacillus plantarum* (Table S1). Of particular interest are some strains of the major commensal of the oral mucosa and oropharyngeal tract, *Strep. salivarius*,^1,4,24^ which possess three distinct SRRPs – SrpA, SrpB and SrpC (Table S1). It should be noted that although many strains of *Strep. salivarius, Strep. vestibularis, Strep. mitis, Strep. oralis* and *Strep. cristatus* are classed as commensals, some have pathogenic potential, having been associated with diseases such as endocarditis or isolated clinically in cases of meningitis, ureolytic bacteremia, spinal operation infections and in immunocompromised situations.^25–31^ In addition, recent bioinformatics analysis of 58 genome-sequenced *L. reuteri* strains showed that homologues of SRRP (and the corresponding linked aSec gene cluster) were mostly found in rodent and pig isolates of *L. reuteri* and absent from strains of human origin.^18^

Our detailed bioinformatics analysis also identified strains that appeared to possess only an incomplete aSec gene cluster, a SRRP that lacked a C-terminal cell wall anchor (possibly the result of a pseudogene, although still capable of exporting a SRRP extracellularly) or an obvious pseudo-SRRP whose domains were encoded by at least two adjacent ORFs (Table S1). These include strains of *Streptococcus thoraltensis, Lactobacillus gasseri, Lactobacillus rhamnosus, Lactobacillus murinus, Lactobacillus nagelii* and *Lactobacillus mucosae*, a species closely related to *L. reuteri*. Interestingly, *L. mucosae* LM1 appeared to have two SecA2/Y2 accessory gene clusters (LBLM1_RS03980-_RS04070 and LBLM1_RS11555-_RS04660), both with associated pseudo-SRRPs, although the latter cluster does not possess any glycosyltransferase (*gtf*) genes. A similar organisation occurs in *Streptococcus* sp. strain DD12^32^ having two SecA2/Y2 gene clusters STRDD12_00537-_00544 and STRDD12_00642-_00656, with associated SRRP and/or pseudo-SRRP genes with the former cluster lacking any *gtf* genes (Table S1).

## Domain organization of SRRPs

### Overall organization

SRRPs are composed of distinct subdomains: an extended atypical signal sequence peptide of around 90 aa at the N-terminus, followed by an AST domain, a short SRR-1 region, a BR, a second and much larger SRR-2, and a LPTXG cell wall anchoring motif.^1,19,20^ The AST domain, required for efficient targeting of the SRRP to the aSec machinery,^20^ is rich in alanine, serine and threonine residues (30–50%) and is typically 30–40 aa long although much shorter AST sequences occur in some SRRPs (Table S2). The SRR domains are typically composed of alternating serine residues, most frequently separated by an alanine, valine or threonine, but other residues such as glutamate, methionine, and leucine can also be found. SRR-2 shows high variability in terms of the nature and number of repeats between species and strains, resulting in the large diversity of sizes occurring in the SRRP family. It has been suggested that the number of repeats in the SRR-2 domains may have evolved to enable the binding region to reach out across the cell surface to mediate attachment.^1^

Table S2 illustrates the domain organization of a range of SRRPs from commensal and pathogenic bacteria. Although not an exhaustive list, examples are included from most of the species that have an aSec and contain at least one SRRP or pseudo-SRRP. Pseudogenes were included in this analysis because many appear capable of expressing a secreted protein that lacks only the LPXTG cell wall anchor motif so that the protein produced may still have a biological function although it would not be covalently attached to the cell surface. The aSec-specific secretion signal ranged in length from 55 aa of the SRRP from *L. fructivorans* to 101 aa of the SRRPs from *L. salivarius*. SRRPs from one or more species can have a predominance of the same SRR-2 motif but this does not apply to all SRRPs from the same species or even SRRPs from the same strain if multiple SRRPs are present, suggesting that these SRR-2 sequences evolved independently or had been acquired via horizontal transfer from other species and then been subject to evolutionary drift (Table S2). For example, the SRRPs from *L. reuteri* rodent strain 100–23C (SRRP_100–23_) and of pig strain ATCC 53608 (SRRP_53608_) display distinct SRR-2 repeating patterns (Table S2). The 10-aa serine-rich motif or repeat (SRR) ‘SLSNSVSMSE’ occurs 91 times in the SRR-2 domain of SRRP_100–23_ and is interspersed with another 10 repeats with slight sequence variations (Table S2). The ‘SLSNSVSMSE’ repeat motif also occurs 10 times in the SRR-2 domain of SRRP_53608_ along with another 15 repeats of differing sequence and is found in several other SRRPs from pig, rodent and sourdough isolates of *L. reuteri* and from strains of *L. johnsonii*. Similar repeating patterns ranging in size from 10 to 20 aa of alternating serine residues but with different sequences are also found in the SRR-2 domains of the other SecA2/Y2-secreted proteins, including those from pathogenic streptococci and staphylococci (Table S2). In contrast to other Gram-positive bacteria which have a unique SRR glycoprotein-encoding gene (see above), *Strep. salivarius* expresses three large and glycosylated surface-exposed proteins – SrpA, SrpB, and SrpC – that show characteristics of SRR glycoproteins and are secreted through an aSec system.

### Binding domains

**Figure 3.**
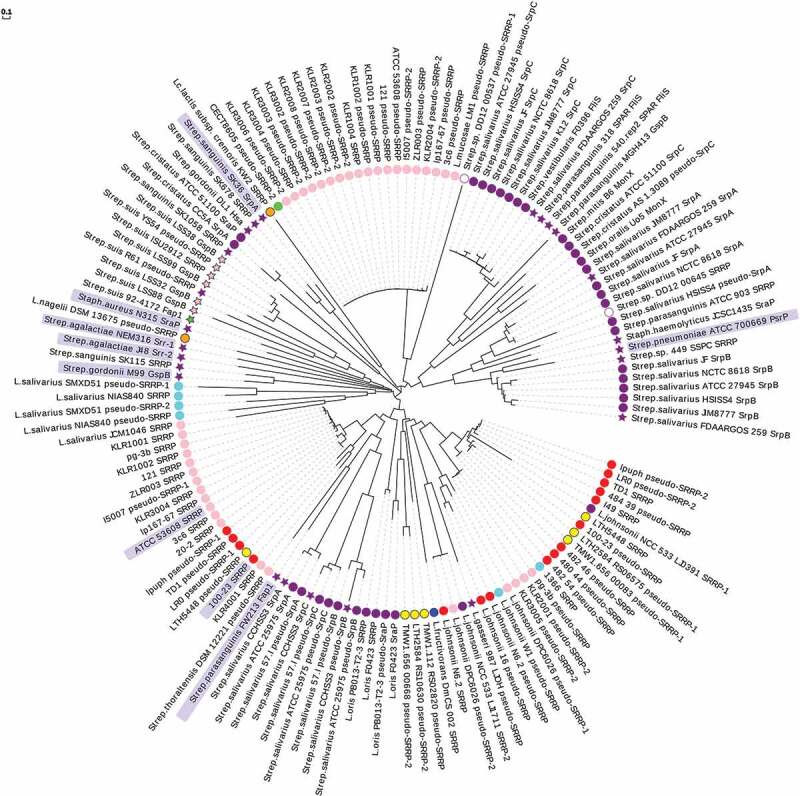
Circular phylogram representation of SRRP-BR domains from commensal and pathogenic bacteria. A MUSCLE multiple sequence alignment^33^ was carried out in MEGA-X^34^ using BR domain aa sequences from a total of 108 commensal-associated SRRPs and 32 pathogen/clinical-associated SRRPs. A guide tree was generated from the second iteration and a Maximum Likelihood phylogenetic tree displayed as a circular phylogram with EvolView software (http://www.evolgenius.info/evolview/).^35^ SRRP-BRs are displayed as follows: circle, from a commensal or non-pathogenic strain; star, from a pathogenic or clinical isolate. Host or source origins of strains are indicated as follows: pink, porcine; red, rodent; purple, human; aqua, avian; lime, bovine; blue, insect; yellow, sourdough; orange, other fermented food or drink; white, chimpanzee fruit residues. BRs shown with only a strain name are all from *L. reuteri*. The entries for which a SRRP-BR crystal structure is available are shaded in a light blue box. The scale bar represents the branch length expressed as the number of aa substitutions per site.

The sequence analysis of SRRP-BRs in lactobacilli genomes was reported previously.^18^ Here the analysis was extended to 140 SRRP-BR domains (covering 108 commensal-associated SRRPs and 32 pathogen/clinical-associated SRRPs) using a MUSCLE multiple sequence alignment^33,34^ and generating a Maximum Likelihood tree displayed as a circular phylogram^35^ (Figure 3). In most cases, SRRPs from commensal bacteria shared low homology in the circular diagram with the well-characterized SRRPs of pathogens – Fap1, GspB, Hsa, PsrP, SraP, SrpA, Srr-1, and Srr-2. This was confirmed in pairwise global alignments of the “pathogenic” SRRP-BRs with selected BR sequences from SRRPs of *L. reuteri* and SrpA/SrpC from *Strep. salivarius* JIM8777 giving aa % identity and aa % similarity values generally <20% and <30%, respectively (Table S3A&B). Specifically, SRRP_53608-_BR shared only 16.8% aa identity and 27.5% aa similarity with *Strep. gordonii* GspB-BR and 13.0% aa identity and 25.0% aa similarity with *Strep. parasanguinis* Fap1-NRα (Table S3A&B). The *Strep. agalactiae* Srr-1 adhesin that includes the K4 sub-domain implicated in the binding to cytokeratin 4 (CK4) showed 16.7% aa identity and 30.3% aa similarity to SRRP_53608_-BR (Table S3A&B). In a few instances, BRs from pathogenic or clinical isolates showed homology to SRRP-BR domains from commensal strains as shown for the BRs from PsrP of *Strep. pneumoniae* and SraP from *Staph. haemolyticus* with six *Strep. salivarius* SrpBs (Figure 3). This was further confirmed by pairwise global alignment with aa % identity values of 46.5% and 35.2% and aa % similarity values of 59.9% and 42.5% for PsrP and SraP, respectively, with SrpB from *Strep. salivarius* JIM8777 (Table S3A&B). Closer examination of the region of homology between the PsrP-BR and SrpB-BR (Figure S1A) revealed that it included the PsrP binding region to keratin 10 (KRT10),^36^ a 71 aa region with 52.1% aa identity and 64.8% aa similarity to the corresponding region in SrpB (Figure S1B) that was also present in the five other members of the SrpB cluster (Figure S1C). This suggests that *Strep. salivarius* SrpB could bind to keratin expressed by the different epithelial cell lines to which this bacterial species is known to bind.^14^

The circular phylogram further showed some host-specific clades for *L. reuteri* BRs (Figure 3). Three major clusters were evident – one group consisting mainly of SRRPs from porcine isolates that included strain ATCC 53608 but also rodent isolate 100–23 (with SRRP_53608_-BR and SRRP_100–23_-BR sharing 44.8% aa identity and 61.5% aa similarity in a pairwise global alignment) (Table S3A&B), a second group mainly of pseudo-SRRPs from rodent and sourdough strains and a third group of pseudo-SRRPs of porcine origin. Interestingly, BRs from *Strep. salivarius* were divided into three main groups – the first comprising SrpA and SrpB from strains JIM8777, JF, ATCC 27945, NCTC 8618, FDAARGOS 259 and HSISS4, a second containing SrpC from the same group of six strains and a third group of SrpA, SrpB and SrpC from strains ATCC 25975, CCHSS3 and 57.I. In *Strep. salivarius* genomes, SrpC is often annotated as the flagellin-specific chaperone, FliS, and in this analysis three FliS-BRs from two other streptococcal species clustered with the group of six SrpCs from *Strep. salivarius* (Figure 3).

Although originally termed the “basic region” in SRRPs first studied from pathogenic bacteria because of the high isoelectric point (pI)^20^ of the binding region, it has become evident in recent years that the corresponding region in many other SRRPs has a net acidic pI.^1,11^ In the present bioinformatics analysis of SRRP amino acid sequences, the majority of BRs (79%) showed a net “strong acidic” pI (arbitrarily set as ranging between pH 4.00–5.55) (Table S4). A few BRs (17%), although still net acidic, showed a pI value between 5.55 and 7.00 (classed as “weak acidic”); these include mainly BRs from SrpC and pseudo-SrpC from *Strep. salivarius* strains along with FliS from *Strep. parasanguinis* and *Strep. vestibularis* strains and SRRPs from *Strep. cristatus*. Only a minority of BRs examined (4%) had a net basic pI (>8.00) with five out of six being from SRRPs of pathogenic strains (such as GspB and Hsa from *Strep. gordonii*, PsrP from *Strep. pneumoniae*, a SRRP from *Strep. sanguinis* and a SraP from *Strep. haemolyticus*). The calculated pI is an estimation of the average electrostatic charge over the entire BR domain and is therefore not predictive of BR function. However, it is worth noting that pH can influence BR structural conformation and binding specificity, as observed for *L. reuteri* SRRPs^18^ and *Strep. parasanguinis* Fap1,^37^ so that information on BR pI, especially around the binding site(s), may inform on the types of ligands recognized by SRRPs under particular pH conditions.

In addition to the typical BRs, some SRRPs from commensal and pathogenic bacteria contain other putative binding regions that could modulate or enhance binding. For example, the gene encoding SrpA in *Strep. salivarius* genomes contains a varying number of repeat motifs annotated as mucin-binding protein (MucBP) located on either side of the SRR-2 domain (Figure S2A). Some SRRPs from other streptococci, including the *Strep. salivarius* clinical isolate CCHSS3^38^ and commensal isolate 57.I,^39^ contain unusually long BR domains that include an L-type lectin region,^40^ a single Rib/alpha/Esp surface antigen repeat^41^ and a hyperosmolarity resistance protein Ebh (N-terminal domain) region^42^ (Table S2). The SRRPs found in many strains of the pathogenic species *Streptococcus suis* also have an exceptionally large BR domain that can contain motifs such as a bacterial group 3 immunoglobulin (Ig)-like domain (Figure S2B) or a FlgD Tudor-like domain (Table S2). These putative binding domains in addition to the well-characterized BR domains may contribute to the binding of SRRPs to a variety of ligands.

## Glycosylation of SRRPs

### SRRP glycosylation pathways

The aSec system contains the necessary genes encoding proteins that facilitate the expression, glycosylation and subsequent secretion of SRRPs.^1^ Interestingly, glycosylation of SRRP appears to promote secretion of the adhesin through the SecA2/Y2 system, and inhibit its export through the canonical SecA system.^43^

The best-studied examples of SecA2/SecY2-mediated glycosylation systems are from pathogenic *Strep. parasanguinis, Strep. pneumoniae, Strep. gordonii, Strep. agalactiae*, and *Staphylococcus aureus*.^44–46^ In all cases, the glycosylation process is initiated by a two-protein glycosyltransferase complex, consisting of GtfA and GtfB that mediate the addition of *N*-acetylglucosamine (GlcNAc) to serine and threonine residues within the SRR domains of the adhesins. These enzymes interact with the acceptor SRRP and with each other through a conserved domain DUF1975 and mediate the addition of the reducing GlcNAc. GtfA acts as a glycosyltransferase (GT), whereas GtfB interacts with the acceptor protein as a chaperone.^47,48^ In certain species, such as *Staph. aureus*, these are the only GTs encoded in the aSecA cluster, and the glycan is not further extended.^8,10^ In bacteria that express additional GTs, GtfC, formerly annotated as nucleotide sugar synthase (Nss), catalyzes the second glycosylation step, in most cases by adding a glucose moiety.^44,45,49,50^ The third glycosylation step is mediated by a domain of unknown function (DUF), DUF1792, that is present in a bifunctional enzyme (dGT1)^45,50,51^ and has been shown to adopt a GT-D fold.^51^ The dGT1 enzyme also harbors a GT-A domain which has been shown to either generate a branching point as in Fap1 with the addition of GlcNAc,^44^ or linearly extend the glycan as in PsrP with the addition of either a Glc or Gal residue.^45^ GTs involved in the glycosylation of PsrP exhibit functional redundancy, as more than one GT can mediate the third and fourth glycosylation steps.^45^ Additional GTs are in some cases involved in the further elongation of the glycan structures.^44,45^ While the genes encoding GtfA and GtfB are conserved in all aSec loci identified to date, the number of genes encoding the additional GTs involved in the glycosylation of SRRPs varies between species, resulting in a range of glycan structures.

Furthermore, previous work on the glycosylation of GspB from *Strep. gordonii* M99 showed that additional heterogeneity is introduced by the *O-*acetylation of GlcNAc residues by Asp2.^52,53^ This modification has also been identified in Srr-1 from *Strep. agalactiae* H36B.^7^ The catalytic residues of Asp2 identified by Seepersaud et al. (2012)^53^ are conserved in *Strep. gordonii* M99, *L. reuteri* 100–23, *L. reuteri* ATCC 53608 and *Strep. salivarius* JIM8777, suggesting that Asp2 may also perform the same reaction in these organisms.

**Figure 4.**
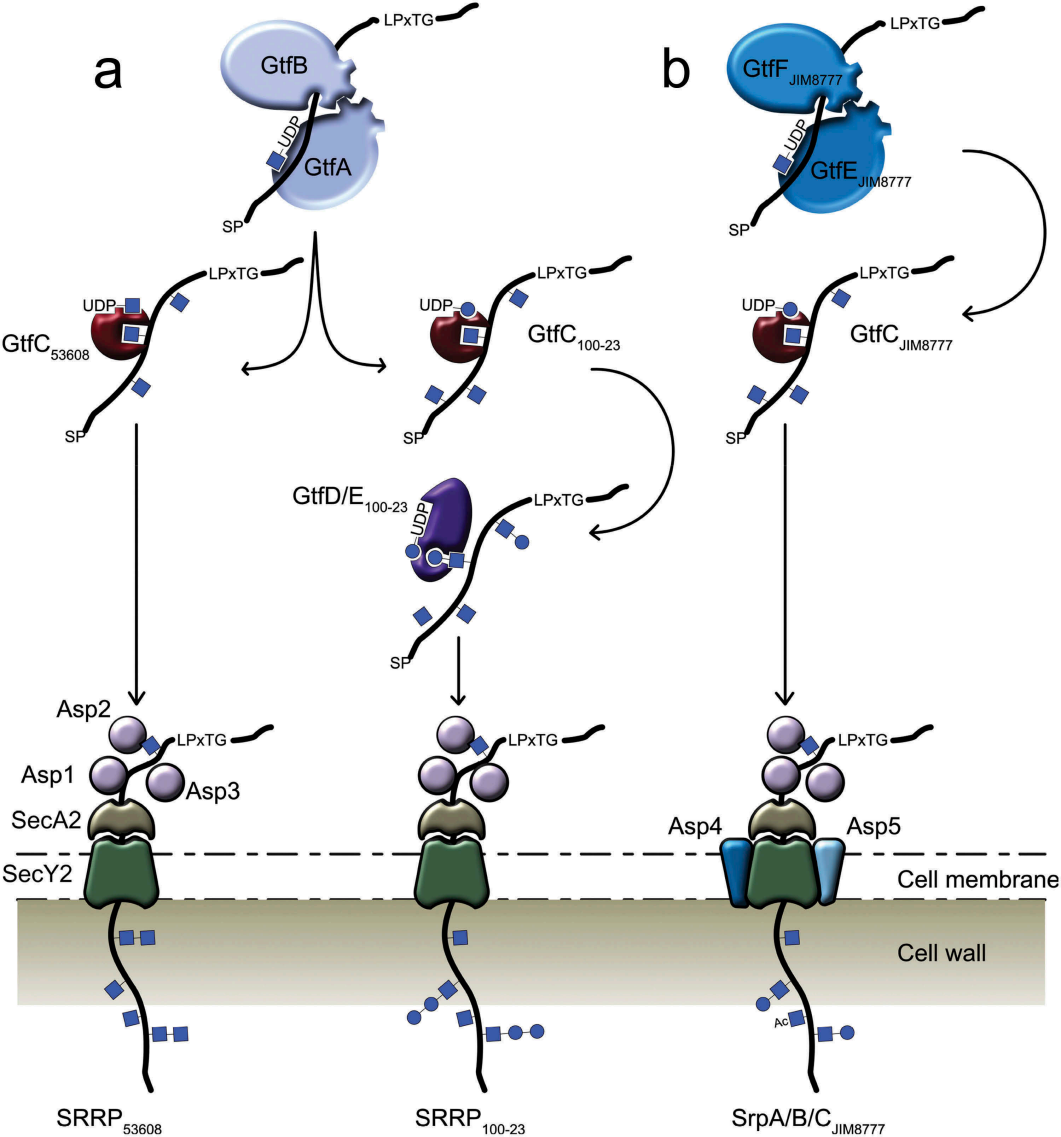
SRRP glycosylation mechanisms in gut commensal bacteria. (a) SRRP glycosylation in *L. reuteri* strains ATCC 53608 and 100–23. The GtfA/B complex initiates the glycosylation of the *L. reuteri* SRRP with GlcNAc residues, while GtfC extends the glycans with either GlcNAc (GtfC_53608_) or Glc (GtfC_100–23_). The glycosylated SRRP_53608_ is then secreted through the aSec system, whereas the SRRP_100–23_ is further extended by GtfD and/or GtfE before secretion. (b) SRRP glycosylation in *Strep. salivarius* strain JIM8777. SrpA, SrpB, and SrpC glycosylation are mediated by the GtfE/F complex, GTs encoded by a genetic locus unlinked to the aSec genomic island. After extension and acetylation of the glycans by other enzymes in the aSec system, the adhesins are secreted through the SecA2/Y2 channel. Blue circle: glucose, blue square: GlcNAc, SP: signal peptide.

In *L. reuteri*, the intact aSec cluster has mostly been found in strains of murine or porcine origin, and it appears to be absent from strains of human origin (see above). In addition to the SecA2 and SecY2 translocases and the accessory secretion-associated proteins Asp1-3, the *L. reuteri* ATCC 53608 SecA2/Y2 glycosylation system contains genes encoding the priming GtfA_53608_ and GtfB_53608_, and a gene encoding GtfC_53608_ whereas in *L. reuteri* 100-23C, the SecA2/Y2 cluster includes eight genes encoding predicted GTs, including GtfA_100-23_, GtfB_100-23_, and GtfC_100-23_ (Figure 2).^16,18,23^ A recent study demonstrated that GtfA/B are involved in GlcNAc attachment to SRRP_100-23_ and SRRP_53608_ while GtfC_53608_ extends the chain with a GlcNAc residue and GtfC_100-23_ with Glc^15^ (Figure 4). To date, all characterized GtfCs have been shown to add a Glc residue onto the GlcNAc core, therefore this was the first report of a GtfC from the SecA2/Y2 system showing ligand specificity to UDP-GlcNAc.^15^ The specificity of *L. reuteri* GtfC_53608_ was further supported by Differential Scanning Fluorimetry (DSF) and Saturation Transfer Difference (STD) NMR analyses, showing a preference for UDP-GlcNAc, in line with the MS/GC-MS analyses.^15^

Glycosylation of the three SRRPs of *Strep. salivarius* JIM8777 follows a slightly distinct process. Despite the presence of genes encoding putative GtfA and GtfB, the first glycosylation appears to be unusually carried out by two genetically linked GTs, GtfE and GtfF, encoded outside of the *secA2/Y2* locus^14^ (Figure 4). In addition, genes encoding a putative GtfC and a putative dGT1 are present in the aSec cluster, suggesting a subsequent glycosylation pathway similar to that of SRRP_100-23_ from *L. reuteri* 100-23.^15^

### Glycosylation profile of SRRPs

SRRPs from pathogenic *Streptococcus* and *Staphylococcus* species carry a range of different glycan structures (Table 1), reflecting differences in the organization of the SecA2/Y2 accessory cluster of these strains.

**Table 1.** Glycan structures identified on SRRPs from different microorganisms.

SRRP	Origin	Glycans		
Fap1	*Strep. parasanguinis* FW213	Glc1-3GlcNAc1-(Rha1-3Glc1)-2,6Glc1-6GlcNAc-^51^		
GspB	*Strep. gordonii* M99	Glc-Glc-GlcNAc-^50^ *O*-AcGlcNAc-^50^		
PsrP	*Strep. pneumoniae* TIGR4	Gal-Glc-Glc-GlcNAc-^45^ Glc-Glc-Glc-GlcNAc-^45^ Gal-Gal-Glc-GlcNAc-^45^ Glc-Gal-Glc-GlcNAc-^45^		
SraP	*Staph. aureus* COL	GlcNAc-^10^		
Srr-1	*Strep. agalactiae* H36B	Contains HexNAc, *O*-AcHexNAc, Hex^7^		ND*
Srr-2	*Strep. agalactiae* COH1	Contains GlcNAc, Glc^49^	ND*	
SrpA SrpB SrpC	*Strep. salivarius* JIM8777	Hex-GlcNAc-^14^ *O*-AcHexNAc-^14^ GlcNAc-^14^		
SRRP_100-23_	*L. reuteri* 100-23	Glc-Glc-GlcNAc-^15^ Gal-Glc-GlcNAc-^15^ GlcNAc-^15^		
SRRP_53608_	*L. reuteri* ATCC 53608	GlcNAcα-^15^ GlcNAcβ1-6GlcNAcα-^15^		

*ND: Not determined; PR: Protein; Ac; *O-*acetyl-; Monosaccharide symbols follow the Symbol Nomenclature for Glycans system.^54^ Key: glucose (blue circle), GlcNAc (blue square), rhamnose (green triangle), galactose (yellow circle), hexose (white circle).

In the commensal *Strep. salivarius* JIM8777 strain, all three SRRP substrates of the SecA2/Y2 system, SrpA, SrpB, and SrpC were found to be glycosylated.^14^ LC‐MS/MS analysis revealed the presence of hexose (Hex), *N*‐acetylhexosamine (HexNAc) and *O*‐acetyl‐*N*‐acetylhexosamine (*O*‐AcHexNAc) linked to peptides in both SRR-1 and SRR-2 domains (Table 1). The SRR-1 domain showed high glycosylation diversity with 24 different glycan combinations whereas HexNAc or *O*‐AcHexNAc modified peptides were found in the SRR-2 domain, with all serine and threonine residues of the C‐terminal SRR-2 domain being glycosylated. Hex residues were only present on peptides carrying at least three HexNAc residues, suggesting that HexNAc modification is a prerequisite for Hex glycosylation (Figure 4). Sequence homology suggested glycosylation of these regions in all three *Strep. salivarius* JIM8777 SRRPs. Furthermore, MS analysis of *Strep. salivarius* SrpA, SrpB, and SrpC identified *O*-acetylated GlcNAc residues^14^, supporting Asp2 activity (see above).

The glycosylation profile of SRRPs from *L. reuteri* was recently determined using a combination of bioinformatics analysis, lectin screening, LC-MS-based sugar nucleotide profiling, MALDI-ToF, and GC-MS analyses. This study showed that the *L. reuteri* ATCC 53608 and 100-23C strains were capable of performing protein glycosylation and that SRRP_100-23_ and SRRP_53608_ were glycosylated with Hex-Hex-HexNAc and di-HexNAc moieties, respectively. Following *in vivo* glycoengineering in *E. coli*, NMR analysis and enzymatic treatment further showed that SRRP_53608_ was glycosylated with GlcNAcβ(1→6)-GlcNAcα moieties. Together, it was suggested that SRRP_100-23_ is glycosylated with GlcNAc and Hex-Glc-GlcNAc whereas SRRP_53608_ is glycosylated with GlcNAc and di-GlcNAc moieties^15^ (Figure 4) (Table 1). Although both strains encode a predicted Asp2, *O*-acetylation could not be confirmed biochemically due to the conditions used in the MS analysis.^15^ The number of GTs in the *L. reuteri* 100-23C SecA2/Y2 cluster exceeds the number of sugars on SRRP_100-23_, as also reported for some streptococcal SecA2/Y2 systems.^14,45^ To date, there is no generic explanation for the presence of additional genes encoding GTs in the genomes of these strains. In some cases, gene duplication is observed, which may lead to functional redundancy, whereas insertion of genetic elements into genes encoding GTs may lead to gene inactivation. A defective glycosylation of SRRPs in pathogenic bacteria led to impaired binding of the respective bacteria onto model substrates and reduced virulence in mouse models.^1,55,56^ The glycosylation of SRRPs in *Lactobacillus* species, as demonstrated for *L. reuteri* strains, is likely to impact on the adhesion capacity of these strains.

## Functional and structural properties of SRRPs

Bacterial attachment to host surfaces is a pivotal event in the biological and infectious processes of both commensal and pathogenic bacteria, respectively. The role of SRRPs in Gram-positive bacterial pathogenesis has been investigated extensively (see Lizcano et al., 2012^1^ for a review). *Streptococcus* and *Staphylococcus* SRRPs contribute towards a wide range of diseases including sub-acute bacterial endocarditis, community-acquired pneumonia, and meningitis.^57,58^ It is believed that the main function of the SRR domains is to form a rigid, *O*-glycosylated, rod-like structure to extend the N-terminal BR domain out from the cell surface to facilitate efficient binding.^1^,^70^70.^^ Crystal structures of seven SRRP-BRs have been reported for Gram-positive pathogens to date, highlighting a relationship between their structural folds and binding ligands (Table 2). These include *Strep. parasanguinis* Fap1 (2KUB and 2X12),^70^ *Strep. gordonii* GspB (3QC5/6),^71^ *Strep. sanguinis* SrpA (5EQ2),^67^ Srr-1 and Srr-2 paralogues of *Strep. agalactiae* (4MBO/R),^68,72^ *Staph. aureus* SraP (4M0(0–3))^65^ and *Strep. pneumoniae* PsrP (3ZGH/I).^36^ For example, Srr-1, Srr-2, and PsrP each adopt variations of the DEv-IgG fold^36,68,73^ and mediate binding to proteins with long α-helical folds, with Srr-1 binding to cytokeratin-4;^69,74,75^ Srr-1 and Srr-2 binding to fibrinogen Aα,^2,68,76–78^ and PsrP adhering to cytokeratin-10,^13^ as well as DNA.^12,36^ Other SRRP-BRs are composed of two or more domain folds and include – from N- to C-terminus – the helical and CnaA folds for Fap1,^70^ CnaA, siglec and ‘unique’ subdomains for GspB,^71^ siglec and ‘unique’ subdomains for SrpA,^67^ and a legume lectin-like fold, a β-grasp fold and two eukaryotic cadherin-like modules for SraP.^65^ The GspB, Hsa, and SrpA homologs from *Strep. gordonii* and *Strep. sanguinis* species bind to sialoglycan ligands on microarrays and platelets.^63,79^ Hsa binds to both α(2–3) sialyllactosamine and sialyl-T antigen, whereas GspB binds only to the latter.^59,71,79–85^ SraP specifically recognizes N-acetylneuraminic acid.^79,86^ Recently, a novel SRRP, SssP1, associated with a SecA2/Y2 gene cluster has been identified in strain CZ130302 of *Strep. suis*, an important Gram-positive pathogen in the swine industry and emerging zoonotic pathogen for humans. SssP1 participates in adhesion to host cells including HEp-2 and human brain microvessel endothelial cells (HBMECs) and contributes to *Strep. suis* virulence. Although SssP1 lacks an LPXTG cell wall anchor which may classify it as a pseudo-SRRP, it contains additional N3 and SRR-3 domains at its C-terminus which may play a role in tethering it to the bacterial cell surface. The SssP1-BR, like those from several other *Strep. suis* SRRPs, contains two bacterial group 3 Ig-like domains (Figure S1B). Recombinant SssP1 proteins that contained these Ig-like-1 and Ig-like-2 domains, respectively, were shown to be involved in the binding to sialic acids^11^ (see Table 2).

**Table 2.** Binding substrates of SRRPs from different microorganisms.

SRRP name	Origin	Binding Substrate
Fap1	*Strep. parasanguinis* FW213	Undetermined saliva components^3^
GspB	*Strep. gordonii* M99	Human salivary proteins, sialyl-T antigen, glycoprotein Ibα^59-61^
Hsa	*Strep. gordonii* DL1	Sialyl-T antigen, α(2,3) sialyllactosamine, sialic acid-containing MUC7, human salivary proteins, glycoprotein Ibα^59,61,62^
SRRP_SK1_	*Strep. sanguinis* SK1	Sialyl-T antigen, sialyl-Lewis X, α(2,3) sialyllactosamine^63^
SRRP_NCTC10712_	*Strep. sanguinis* NCTC10712	Sialyl-T antigen, sialyl-Lewis X, α(2,3) sialyllactosamine^63^
SRRP_SK678_	*Strep. sanguinis* SK678	Sialyl-Lewis X, α(2,3) sialyllactosamine^63^
SRRP_SF100_	*Strep. mitis* SF100	Sialyl-T antigen^63^
PsrP	*Strep. pneumoniae* TIGR4	Keratin-10, BR_PsrP_, extracellular DNA^12,13,64^
SraP	*Staph. aureus* COL	Surface molecules of blood platelets, sialylated structures^65,66^
SrpA	*Strep. sanguinis* SK36	Platelets, sialic acid residues^67^
Srr-1	*Strep. agalactiae* CNCTC 10/84	Platelets, keratin-4, fibrinogen^2,68,69^
Srr-2	*Strep. agalactiae* COH1	Fibrinogen^68^
SssP1	*Strep. suis* CZ130302	HEp-2, human microvessel endothelial cells, sialylated structures^11^
SRRP_100-23_	*L. reuteri* 100-23	Mouse forestomach stratified squamous epithelium^17^
SRRP_53608_	*L. reuteri* ATCC 53608	Porcine gastric mucin, DNA, intestinal epithelium, polygalacturonic acid, rhamnogalacturonan I and chondroitin sulfate A^18^
SrpB	*Strep. salivarius* JIM8777	Epithelial cells (HT-29, A549, FaDu, HEp-2)^14^
SrpC	*Strep. salivarius* JIM8777	Extracellular matrix components (MUC2, fibronectin, MaxGel ECM, laminin, Elastin)^14^

In the gut commensal bacterium *Strep. salivarius*, SrpA, SrpB and SrpC are the main factors underlying its multifaceted adhesion and, their glycosylation plays a major role in host colonization.^14,65^ SrpB and SrpC play complementary adhesive roles involved in several steps of the colonization process: auto-aggregation, biofilm formation and adhesion to a variety of host epithelial cells and components. At least one of the *Strep. salivarius* SRR glycoproteins is important for colonization in mice. A preliminary analysis of the binding targets for these three SRRPs indicated that SrpC bound to mucin MUC2 and to various extracellular matrix proteins whereas SrpB participated in bacterial autoaggregation and was involved in adhesion to various human epithelial cell lines.^14^ The ligands for SrpA have yet to be identified, despite being annotated as having MucBP repeat motifs (Figure S1A), but it was suggested that SrpA could be an adhesin involved in interspecies co-aggregation.^14^ No crystal structure of *Strep. salivarius* SRRP-BRs is currently available (see Table 2).

In *L. reuteri*, the SecA2/Y2 cluster and SRRP in the murine isolate *L. reuteri* 100-23 is crucial for adhesion of the bacteria to the forestomach epithelium of the murine GI tract, as shown by colonization experiments in germ-free mice with *L. reuteri* 100-23 wild-type and mutants.^17^ Mutants lacking the s*ecA2* gene showed defective adhesion, whereas mutants lacking *srrp* showed the most reduced biofilm formation, compared to other putative adhesins tested.^17^ In contrast to all structurally characterized SRRP-BRs reported to date, *L. reuteri* SRRP-BR displays a fold typically adopted by extracellular pectate lyase PelC-like proteins.^18^ The BR crystal structures of SRRP_100-23_ and SRRP_53608_ from *L. reuteri* ATCC 53608, revealed a “β-solenoid” fold comprising β-strands coiled in a repetitive pattern to form a right-handed helix with three parallel β-sheets, which is unique in the SRRP family.^18^

SRRP_53608_-BR bound to host epithelial cells, mucins, and DNA at neutral pH and recognized polygalacturonic acid (PGA), rhamnogalacturonan I or chondroitin sulfate A at acidic pH. Mutagenesis confirmed the role of the BR putative binding site in the interaction of SRRP_53608_-BR with PGA. Long molecular dynamics (MD) simulations showed that SRRP_53608_ undergoes a pH-dependent conformational change that would mediate the different binding profiles observed. A Dali search revealed that the structure of *L. reuteri* SRRP-BRs also shows similarity to some extracellular adhesive proteins with PelC-like folds in pathogens, such as pertactins or bacteriophage tail-spike proteins.^18^

**Figure 5.**
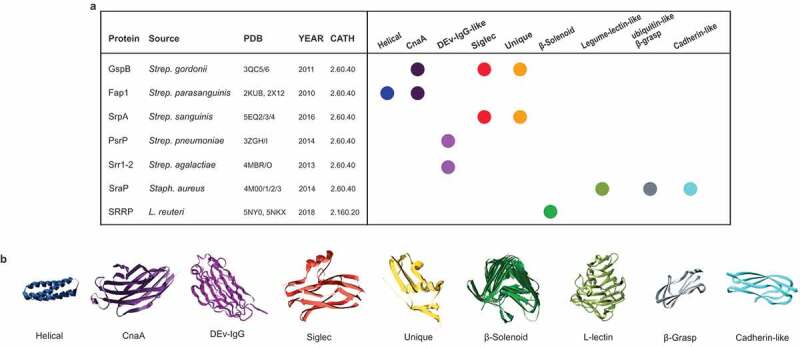
Structural repertoire of the SRRP binding regions. (a) Summary of the domains found in the available structures of SRRPs. For each protein, we report the name, the source organism, the PDB code of the available structures, the year of the publication of the structure and the CATH code. On the right, a schematic representation is included of the structural domains present in the proteins. (b) Example of three-dimensional structure of each domain found in the SRRP-BRs.

A summary of the structural organization and fold of SRRP-BRs structurally characterized to date is provided in Figure 5, where we also report the CATH^87^ family of each protein. All the proteins, excluding the *L. reuteri* ones, belong to the “Immunoglobulin-like” family. The structures of Srr-1 and Srr-2 (*Strep. agalactiae*), while structurally in the same CATH category, do not share specific domains with the other SRRPs of the list, and share a higher structural similarity with clumping factor A of *Staph. aureus* (Newman strain). The structure of *L. reuteri* SRRP-BRs is radically different (CATH 2.160.20, Pectate Lyase C-like) to all other SRRP-BRs from pathogenic bacteria for which a crystal structure is available, perhaps reflecting a different role in gut-commensal interactions. Interestingly, a sequence similarity search revealed an uncharacterized non-SRR protein (WP_003669640.1) in *L. reuteri* human strain DSM 20016 (lacking the aSec pathway), that is likely to share the same β-solenoid organization (according to homology modelling) and could be evolutionary related to the SRRPs found in *L. reuteri* strains 100-23 and ATCC 53608, isolated from rat and pig, respectively. The variety of domains found in the binding region of proteins from different organisms, suggests that the evolutionary adaptation to different niches could require domain shuffling, eventually acquiring existing domains from other proteins. The diversity in SRRP-BR structures is reflected in their primary amino acid sequences, as illustrated in Figure 3.

SRR glycoproteins from *L. reuteri* strains may contribute to the mechanisms underpinning *L. reuteri* adaptation to their vertebrate hosts. In addition, the presence of complete SecA2/Y2 clusters with an intact SRRP in the genomes of other *Lactobacillus* species (see above), suggest a common role of SRR glycoproteins in adhesion to host epithelia, which may be related to the ecological context of these strains (see Duar et al., 2017^88^ for a review).

## Conclusions and future directions

Bacterial adhesion is a critical step for colonization of the host. Cell surface proteins (adhesins or lectins) mediate attachment to host cells, initiate colonization and define a bacterial cell and tissue tropism by interacting with host proteins or glycoconjugates. SRRPs expressed by Gram-positive bacteria are important mediators of the interaction between streptococci and staphylococci with the host tissues. SRRPs show a high sequence diversity, which is reflected by the modularity and structural folds of their binding domains. The finding that SRRPs occur in non-pathogenic bacteria expands the role played by this important family of glycosylated adhesins in mediating gut microbiota–host interactions. Knowledge of the structure and function of SRRPs from gut microbes may be used for the selection of probiotic strains targeting different vertebrate hosts. Another potential application is to explore these adhesion/biofilm formation properties to compete with clinically important pathogens, reduce infection and combat antimicrobial resistance. However, more work is needed to ascertain the exact nature of the receptors of these adhesins *in vivo*.

Protein glycosylation is a major feature of SRRPs. These adhesins harbor different glycan structures reflecting differences in the organization of the SecA2/Y2 accessory cluster of these strains, and in their binding capacity. Investigating the co-evolution of the SRR glycoproteins with their specialized glycan modifying and export systems is warranted to gain novel evolutionary insights into host/niche-specific adaptation. This potential requirement for the coupling of glycosylation and secretion has been proposed as a mechanism to ensure that the adhesin is optimally modified for binding. Whether the post-translational modification and transport of SRRPs follow distinct processes and the impact of protein glycosylation on the biological role of SRRP adhesins are important research questions which remain to be addressed. The discovery that glycosylation is strain-specific provides a molecular track to explore the impact of protein glycosylation in biofilm formation, adhesion, and colonization of gut symbionts. Furthermore, novel information on the cellular pathways leading to the glycosylation of SRRPs expands the range of glycosyltransferase specificities and potential glycoengineering applications for the recombinant production of glycoprotein conjugates in different cell types.
