# Supplementary material for: Serine-rich repeat proteins from gut microbes
Source: Gut Microbes. 2019 Apr 29;11(1):102–17. doi: 10.1080/19490976.2019.1602428 (PMC6973325; doi:10.1080/19490976.2019.1602428)
Supplement: Supplemental Material [file kgmi-11-01-1602428-s001.zip › Supplementary information/Figure S2_A, MucBP & B, Big_3 motifs in SRRPs.docx]

**(A)**

**(i)** *Strep. salivarius* FDAARGOS_259 SrpA with X10 MucBPs (WP_080611899.1):

216 ----------------------------------------------- N2(BR) ---------------------------------------------- 787

788 ----------------------------------------------- SRR-2 ----------------------------------------------- 1286

1287 PKVGEVIITYIRENDGKEIQKPRQDTPNSPYDTPYNTTEEGEKPNTIKTPDGKTYKIVPKGDYPVGKVDGDGHLESSDPIKGKVDKPKSTITYVYK 1382 MucBP-1

1383 EVKGNVYVHYVDTEGKTIKASVTDEKDQPVDKDYDTVVDNRPKEIEFEGKTYELVPAGNYKVGQVDEQGHWTGDDATTGKVIEGDKNVTYVYQLKEQPVQ 1482 MucBP-2

1483 PKGNVYVHYVDTEGKTIKASVTDEKDQPVGKDYDTVVDNRPKEIEFEGKTYELVPAGNYKVGQVDEQGHWTGDDATTGKVIEGDKNVTYVYKLKEQPAQ 1581 MucBP-3

1582 PKGNVYVHYVDENGNTIKASVTDEKDQPVGKDYDTVVDNRPKEIEFQGKTYELVPAGNYKVGQVDEQGHWTGDDATTGKVIEGDKNVTYVYKLKEDPTK 1680 MucBP-4

1681 PKEGDVIITYVDEKGKEIKKPRQDTPNSPYDTPYNTTEEGEKPKTIKTPDGKTYKIVPKGDYPVGKVDGDGHLESSDPIKGKVDKPRSIITYVYK 1775 MucBP-5

1776 EVKGDVYVHYKDTEGNTIKTSVVDEKDQPVDKEYDTVVDNRPKTITTTDGKVYELVPAGNYTVGKVDGQGHLESSDATTGKVIEGRKDVTYIYKLKEQPVQ 1876 MucBP-6

1877 PKGNVYVHYVDENGNTIKASVTDEKDQPVGKDYDTVVDNRPKEIEFEGKTYELVPAGNYKVGQVDEQGHWTGDDATTGKVIEGDKNVTYVYKLKEDPTK 1975 MucBP-7

1976 PKEGDVIITYVDEKGKEIKKPRQDTPNSPYDTPYNTTEEGEKPKTIKTPDGKTYKIVPKGDYPVGKVDGDGHLESSDPTKGKVEKPRSIITYVYK 2070 MucBP-8

2071 EVKGDVYVHYKDTEGNTIKTSVVDEKDQPVDKDYDTVVDNRPKTITTTDGKVYELVPAGNYTVGKVDGQGHLESSDATTGKVIEGRKDVTYIYKLKEQPAQ 2171 MucBP-9

2172 PKGNVYVHYVDENGNTIKQSVTDEFGQPVGKDYDTVIDNRPKTIVTADGKVYELVPQGNYPVGSVDGDGHLTTTDPVTGKVIEGDKNVTYVYKL 2265 MucBP-10

2266 VDTTPEKPVTPTPGKPEQPTPGKPVDPAPKAPAKATPVKPAQEMAQLPNTGEESNVAATAALGLLATASGLALAAKRKKN 2345

**(ii)** *Strep. salivarius* JIM8777 SrpA with X9 MucBPs (CCB95768.1):

216 ----------------------------------------------- N2(BR) ---------------------------------------------- 787

788 ----------------------------------------------- SRR-2 ----------------------------------------------- 1350

1351 PKVGEVIITYIRENDGKEIQKPRQDTPNSPYDTPYNTTEEGEKPNTIKTPDGKTYKIVPKGDYPVGKVDGDGHLESSDPIKGKVDKPKSTITYVYK 1446 MucBP-1

1447 EVKGDVYVHYKDTDGNTIKDDVTDEKDQPVDKDYDTVIDNRPKEIQYNGKTYELVPAGNYNVGKVDEQGHLESSDATTGKVIEGRKDVTYIYKLKEQPAQ 1546 MucBP-2

1547 PKGNVYVHYVDTDGNTIKASVTDEKDQPVGKDYDTVVDNRPKEIEFQGKTYELVPAGNYKVGQVDEQGHWTGDDATTGKVVEGDKNVTYVYKLKEDPTK 1645 MucBP-3

1646 PKEGDVIITYVDEKGKEIKKPRQDTPNSPYDTPYNTTEEGEKPNTIKTPDGKTYKIVPKGDYPVGKVDGDGHLESSDPIKGKVDKPRSIITYVYK 1740 MucBP-4

1741 EVKGDVYVHYKDTEGNTIKTSVVDEKDQPVDKDYDTVVDNRPKTITTTDGKVYELVPAGNYTVGKVDGQGHLESSDATTGKVIEGRKDVTYIYKLKEQPAQ 1841 MucBP-5

1842 PKGNVYVHYVDENGNTIKASVTDEKDQPVGKDYDTVVDNRPKEIEFEGKTYELVPAGNYKVGQVDEQGHWTGDDATTGKVIEGDKNVTYVYKLKEDPTK 1940 MucBP-6

1941 PKEGDVIITYVDEKGKEIKKPRQDTPNSPYDTPYNTTEEGEKPNTIKTPDGKTYKIVPKGDYPVGKVDGDGHLESSDPIKGKVEKPRSIITYVYK 2035 MucBP-7

2036 EVKGDVHVHYKDTEGKTIKTSVVDEKDQPVDKDYDTVVDNRPKTITTTDGKVYELVPAGNYTVGKVDGQGHLESSDATTGKVIEGRKDVTYIYKLKEQPAQ 2136 MucBP-8

2137 PKGNVYVHYVDENGNTIKQSVTDEFGQPVGKDYDTVIDNRPKTIVTADGKVYELVPQGNYPVGSVDGDGHLTTTDPVTGKVIEGDKNVTYVYKL 2230 MucBP-9

2231 VDTTPEKPVTPTPGKPEQPTPGKPVDPAPKAPAKATPVKPAQEMAQLPNTGEESNVAATAALGLLATASGLALAAKRKKTEE 2312

**(iii)** *Strep. salivarius* ATCC 27945 SrpA with X6 MucBPs (NX99_00100: translated 55976-61927nt)^a^:

218 ----------------------------------------------- N2(BR) ---------------------------------------------- 789

790 ----------------------------------------------- SRR-2 ----------------------------------------------- 1316

1317 PKVGEVIITYIRENDGKEIQKPRQDTPNSPYDTPYNTTEEGEKPNTIKTPDGKTYKIVPKGDYPVGKVDGDGHLESSDPIKGKVDKPKSTITYVYK 1412 MucBP-1

1413 EVKGDVYVHYKDTDGNTIKDDVTDEKDQPVDKDYDTVIDNRPKEIQYNGKTYELVPAGNYNVGKVDEQGHLESSDATTGKVIEGRKDVTYIYKLKEQPAQ 1512 MucBP-2

1513 PKGNVYVHYVDTDGNTIKASVTDEKDQPVGKDYDTVVDNRPKEIEFQGKTYELVPAGNYKVGQVDEQGHWTGDDATTGKVVEGDKNVTYVYKLKEDPTK 1611 MucBP-3

1612 PKEGDVIITYVDEKGKEIKKPRQDTPNSPYDTPYNTTEEGEKPNTIKTPDGKTYKIVPKGDYPVGKVDGDGHLESSDPIKGKVEKPRSIITYVYK 1706 MucBP-4

1707 EVKGDVYVHYKDTEGNTIKTSVVDEKDQPVDKDYDTVVDNRPKTITTTDGKVYELVPAGNYTVGKVDGQGHLESSDATTGKVVEGRKDVTYIYKLKEQPAQ 1807 MucBP-5

1808 PKGNVYVHYVDENGNTIKQSVTDEFGQPVGKDYDTVIDNRPKTIVTADGKVYELVPQGNYPVGSVDGDGHLTTTDPVTGKVIEGDKNVTYVYKL 1901 MucBP-6

1902 VDTTPEKPVTPTPGKPEQPTPGKPVDPAPKAPAKATPVKPAQEMAQLPNTGEESNVAATAALGLLATASGLALAAKRKKTEE 1983

**(iv)** *Strep. salivarius* HSISS4 pseudo-SrpA with X6 MucBPs (HSISS4_01309-_01307: translated 1466645-1461427nt)^b^:

216 ----------------------------------------------- N2(BR) ---------------------------------------------- 790

791 ----------------------------------------------- SRR-2 ----------------------------------------------- 1059

1060 PKVGEVIITYIRENDGKEIQKPRQDTPNSPYDTPYNTTEEGEKPNTIKTPDGKTYKIVPKGDYPVGKVDKDGHLESSDPTKGKVEKPRSIVTYVYK 1155 MucBP-1

1156 EVKGDVYVHYKDTEGNTIKTDVTDEKDQPVDKDYDTVVDNRPKEIKYNGKTYELVPAGNYTVGKVDGQGHLESSDATTGKVVEGRKDVTYIYKLKEQPAQ 1255 MucBP-2

1256 PKGNVYVHYVDENGNTIKQSVTDESKQPVDKDYDTVIDNRPKEIEFQGKTYELVPAGNYKVGQVDEQGHWTGDDATTGKVIEGDKNVTYVYKLKEEPVQ 1354 MucBP-3

1355 PKGNVYVHYVDTEGNIIKDSVTDELAQPVGKDYDTVVDNRPKEIEFQGKTYELVPAGNYKVGKVDEQGHWNGDDATTGKVIEGDKNVTYVYQLKEQPAQ 1453 MucBP-4

1454 PKGNVYVHYVDENGNTIKQSVTDESKQPVGKDYDTVVDNRPKEIEFQGKTYELVPAGNYKVGQVDEQGHWTGDDATTGKVIEGDKNVTYIYKLKEQPAQ 1552 MucBP-5

1553 PKGNVYVHYVDENGNTIKQSVTDESKQPVGKHYDTVIDNRPKTIVTADGKVYELVPQGNYPVGKVDGDGHLTTTDPVTGKVIEGDKNVTYVYKL 1646 MucBP-6

1647 VDTTPEKPVTPTPGKPEQPTPGKPVDPAPKAPAKATPVKPAQEMAQLPNTGEESNVAATAALGLLATASGLALAAKRKKTEE 1728

**(v)** *Strep. parasanguinis* ATCC 903 SRRP_903_ with X8 MucBPs (WP_050783544.1):

196 ----------------------------------------------- N2(BR) ---------------------------------------------- 731

732 EKGNVYVHYKDTEGTTIKASVTDENQQPINKHYDTVVDNRPGTIEYNGKTYELVPAGTYTVGQVDSDGHLTTSDPITGSVAKEDKNVTYIYKLKED 827 MucBP-1

828 ----------------------------------------------- SRR-2 ----------------------------------------------- 1278

1279 PKQGEVIITYVDTKGKVIKDPRQDTPNSPYDTPYNTTEEGEKPNTIKTPDGKTYKIVPKGDYPVGKVDEDGHLESSDPIKGKVDKPKSTITYVYQ 1373 MucBP-2

1374 EVSNVYVHYVDTEGNTIKASVTDEKEQPIGKDYDTVVDNRPQTIEFQGKTYELVPAGNYPVGQVDEQGHWTGDDKTTGKVAEKDKNVTYVYQLKQ 1468 MucBP-3

1469 PKGNVYVHYVDVNGNKIKDDVTDEKDQPVGKDYDTVVDNRPSTIEFQGKTYELVPAGNYPVGQVDEQGHWTGDDATTGKVVEGDKNVTYVYKLKEDPTK 1567 MucBP-4

1568 PKEGDVIITYVDEKGKEIQKPRQDTPNSPYDTPYNTTEEGEKPNTIKTPDGKTYKIVPKGDYPVGKVDGDGHLESSDPIKGKVDKPRSIITYVYK 1662 MucBP-5

1663 EVKGDVYVHYKDTEGNTIKDDVTDEKDQPVDKDYDTVVDNRPKEIQYNGKTYELVPAGNYTVGKVDEQGHLESSDATTGKVIEGRKDVTYIYKLKEQPTQ 1762 MucBP-6

1763 PKGSVYVHYKDTEGNTIKESVTDELDQPVGKDYNTVEDNRPQYIRFEGKTYEIVPVGNYTVGKVDTQGHLESTDPTTGKVVEGRKDVTYIYKLVEEPVQ 1861 MucBP-7

1862 PKGNVYVHYVDENGNTIKTSVVDEKDQPVGKDYDTVVDNRPKTITTADGKVYELVPQGNYPVGNVDGEGHLTTTDPTTGKVIEGDKNVTYVYKL 1955 MucBP-8

1956 VKTPNVPTPNTPVPPTPTPNTPVPPTPTPNTPVDPTPNKPMDPTPNTPVDPTPNTPVNPVPEQPAKPAPALEQLPNTGETGSVASALLGAVAGVAGVAALG 2056

2057 SRKKEDEK 2064

**(vi)** *Strep. salivarius* NCTC 8618 SrpA with X8 MucBPs (SSAL8618_07165-_07160: translated 1546119-1540060)^a^:

216 ----------------------------------------------- N2(BR) ---------------------------------------------- 783

784 EGNVYVHYVDTEGKTIKAFVTDEKEQPVGKDYDTVIDNRPQEIEFEGKTYELVPAGNYTVGDVDDQGHLKSTDATTGKVIEGDKHVTYVYKLK 876 MucBP-1

877 EVKGNVYVHYVDTEGNELKPSVTDEENQPVDKDYDTVVDNRPQTIEKDGKTYELVPAGNYKVGQVDSDGHLTSTDATTGKVIEGDKNVTYVYKLKEQ 973 MucBP-2

974 PKGNVYVHYVDVNGNKIKDDVTDEDKQPVDKDYDTVVDNRPKTITTTDGKVYELVPEGNYPVGQVDSQGHWTGDDATTGKVIEGDKNVTYVYKLKEE 1070 MucBP-3

1071 ----------------------------------------------- SRR-2 ----------------------------------------------- 1451

1452 PKVGEVIITYIRENDGKEIQKPRQDTPNSPYDTPYNTTEEGEKPKTIKTPDGKTYKIVPKGDYPVGKVDGDGHLESSDPIKGKVDKPKSTITYVYK 1547 MucBP-4

1548 EVKGNVYVHYVDVNGNKIKDDVTDEKDQPVDKDYDTVIDNRPKEIEFQGKTYELVPAGNYPVGKVDEQGHWNGDDATTGKVVEGDKNVTYVYKLKEDPTK 1647 MucBP-5

1648 PKEGDVIITYVDEKGKEIKKPRQDTPNSPYDTPYNTTEEGEKPKTIKTPDGKTYKIVPKGDYPVGKVDGDGHLESSDPTKGKVEKPRSIITYVYK 1742 MucBP-6

1743 EVKGDVHVHYKDTEGKTIKTSVVDEKDQPVDKDYDTVVDNRPKTITTTDGKVYELVPAGNYPVGKVDGQGHLESSDATTGKVIEGRKDVTYIYKLKEQPAQ 1843 MucBP-7

1844 PKGNVYVHYVDENGNTIKQSVTDEFGQPVGKDYDTVIDNRPKTIVTADGKVYELVPQGNYPVGSVDGDGHLTTTDPVTGKVIEGDKNVTYVYKL 1937 MucBP-8

1938 VDTTPEKPVTPTPGKPEQPTPGKPVDPAPKAPAKATPVKPAQEMAQLPNTGEESNVAATAALGLLATASGLALAAKRKKTEE 2019

**(vii)** *Strep. salivarius* JF SrpA with X10 MucBPs (AMB83116.1):

216 ----------------------------------------------- N2(BR) ---------------------------------------------- 783

784 EGNVYVHYVDTEGKTIKAFVTDEKEQPVGKDYDTVIDNRPQEIEFEGKTYELVPAGNYTVGDVDDQGHLKSTDATTGKVIEGDKHVTYVYKLK 876 MucBP-1

877 EVKGNVYVHYVDTEGNELKPSVTDEENQPVDKDYDTVVDNRPQTIEKDGKTYELVPAGNYKVGQVDSDGHLTSTDATTGKVIEGDKNVTYVYKLKEQ 973 MucBP-2

974 PKGNVYVHYVDVNGNKIKDDVTDEDKQPVDKDYDTVVDNRPKTITTTDGKVYELVPEGNYPVGQVDSQGHWTGDDATTGKVIEGDKNVTYVYKLKEQ 1070 MucBP-3

1071 PKGNVYVHYVDVNGNKIKDDVTDEDKQPVDKDYDTVVDNRPKTITTTDGKVYELVPEGNYPVGQVDSQGHWTGDDATTGKVIEGDKNVTYVYKLKEQ 1167 MucBP-4

1168 PKGNVYVHYVDVNGNKIKDDVTDEDKQPVDKDYDTVVDNRPKTITTTDGKVYELVPEGNYPVGQVDSQGHWTGDDATTGKVIEGDKNVTYVYKLKEE 1264 MucBP-5

1265 ----------------------------------------------- SRR-2 ----------------------------------------------- 1645

1646 PKVGEVIITYIRENDGKEIQKPRQDTPNSPYDTPYNTTEEGEKPKTIKTPDGKTYKIVPKGDYPVGKVDGDGHLESSDPIKGKVDKPKSTITYVYK 1741 MucBP-6

1742 EVKGNVYVHYVDVNGNKIKDDVTDEKDQPVDKDYDTVIDNRPKEIEFQGKTYELVPAGNYPVGKVDEQGHWNGDDATTGKVVEGDKNVTYVYKLKEDPTK 1841 MucBP-7

1842 PKEGDVIITYVDEKGKEIKKPRQDTPNSPYDTPYNTTEEGEKPKTIKTPDGKTYKIVPKGDYPVGKVDGDGHLESSDPTKGKVEKPRSIITYVYK 1936 MucBP-8

1937 EVKGDVHVHYKDTEGKTIKTSVVDEKDQPVDKDYDTVVDNRPKTITTTDGKVYELVPAGNYPVGKVDGQGHLESSDATTGKVIEGRKDVTYIYKLKEQPAQ 2037 MucBP-9

2038 PKGNVYVHYVDENGNTIKQSVTDEFGQPVGKDYDTVIDNRPKTIVTADGKVYELVPQGNYPVGSVDGDGHLTTTDPVTGKVIEGDKNVTYVYKL 2131 MucBP-10

2132 VDTTPEKPVTPTPGKPEQPTPGKPVDPAPKAPAKATPVKPAQEMAQLPNTGEESNVAATAALGLLATASGLALAAKRKKTEE 2213

**(B)**

**(i)** *Strep. suis* CZ130302 SssP1 with X2 Big_3_3 pfam13750 (AWD32147.1):

197 ------------------------------------------------------------- N2(BR) ------------------------------------------------------------- 1518

1519 DVSDLPAATYTVDVEATDSAGTTTTSSFQLTVKDNTPPVITAADRTVDKGAVATYDLRTGVTVRDVEDEANNLTPSVTKIVLKDASTNTVISTITNTSQMTINTSTLDPTKTYTVEVTGSDAEGLTATTS 1648

1649 ------------------------------------------------------------- N2(BR) ------------------------------------------------------------- 2294

2295 DLTNLPAGTYTVSVTATDSTGAVKTATSSITLQENTAPNVTVSDQTIDRGGKTTIDISTGVTVTDTEDDRDPNDSLTTTVTYTVKDSKGSIVYQGSQPNVPVETLVAGIYTVTVAATDAHGAKTEKTYTLKVTDRN 2430

2431 ------------------------------------------------------- SRR-2 / N3 / SRR-3 ------------------------------------------------------- 4647

**(ii)** *Strep. suis* LSS38 GspB with X1 Big_3_3 pfam13750 and X1 partial Big_3_3 (CYV01575.1):

234 --------------------------------------------------------------- N2(BR) ---------------------------------------------------------------- 613

614 ITGTGTPYSTVTLTFSNGAKTTTVVQANGTWTATPPSGAMAASTTVSA 661

662 --------------------------------------------------------------- N2(BR) ---------------------------------------------------------------- 2029

2030 TVDVGNLPAGDYTVRISALDSGGTQTVTGSYPLRINDNTPPVITATDRTVDRSDGSHIDLSTGVTIRDTEDNASGTALTVTYKVVDSKGATVYEGRDPNVASSLLQAGDYTVTVSATDSHGAKTEKSYKLKVTDR 2164

2165 ------------------------------------------------------------ SRR-2 / LPNTG ------------------------------------------------------------ 2603

**(iii)** *Strep. suis* LSS99 GspB with X1 Big_3_3 pfam13750 and X1 partial Big_3_3 (CYV58215.1):

234 --------------------------------------------------------------- N2(BR) ---------------------------------------------------------------- 605

606 ITGTGKAGSTVTLRFSNGTTTTAVVQSNGTWTATPPTGAMATSKTVTAT 654

655 --------------------------------------------------------------- N2(BR) ---------------------------------------------------------------- 1999

2000 TVDVGNLPAGDYTVRISALDSGGTQTVTGSYPLRINDNTPPVITATDRTVDRSDGSHIDLSTGVTIRDTEDNASGTALTVTYKVVDSKGATVYEGRDPNVASSLLQAGDYTVTVSATDSHGAKTEKSYKLKVTDR 2134

2135 ------------------------------------------------------------ SRR-2 / LPNTG ------------------------------------------------------------ 3356

**(iv)** *Strep. suis* ISU2912 SRRP with X1 Big_3_3 pfam13750 (KPA67640.1):

234 ---------------------------------------------------------------- N2(BR) --------------------------------------------------------------- 2192

2193 TVDVGNLLAGDYTVRISALDSGGTQTVTGSYPLRINDNTPPVITATDRTVDRSDGSHIDLSTGVTIRDTEDNASGTALTVTYKVVDSKGATVYEGRDPNVASSLLQAGDYTVTVSATDSHGAKTEKSYKLKVTDR 2327

2328 ------------------------------------------------------------ SRR-2 / LPNTG ------------------------------------------------------------ 3394

**(v)** *Strep. suis* LSS88 GspB with X1 Big_3_3 pfam13750 (CYV65198.1):

233 ---------------------------------------------------------------- N2(BR) --------------------------------------------------------------- 1558

1559 NVNELTAGTYTVNVEATDSVGTTTTSSFQLTVKDNTPPAITGSNRTVDRNDSPTIDISVGVSVTDTEDAAAGVRPSVTYKVVNKNGKVVYKGTNPNVSSGTLLAGVYTVTVTAVDSHGAKTEKSYQLTVTD 1689

1690 ------------------------------------------------------------ SRR-2 / LPNTG ------------------------------------------------------------ 2497

**(vi)** *Strep. suis* YS54 pseudo-SRRP with X1 Big_3_3 pfam13750 (SST61_RS0109470 translated 84457-91718nt):

234 ---------------------------------------------------------------- N2(BR) --------------------------------------------------------------- 2203

2204 TVDVGNLPAGDYMVRISALDSGGTQTVTGNYSLRLNDNTPPVITATDRTVDRSDGSHIDLSTGVTIRDTEDNASGTALTVTYKVVDSKGATVYEGRNPNVASSLLQAGDYTVTVSATDSHGAKTEKSYKLKVTDR 2338

2339 ---------------------------------------------------------------- SRR-2’ --------------------------------------------------------------- 2420

**(vii)** *Strep. suis* LSS32 GspB with X1 Big_3_4 pfam13754 (CYU41319.1):

250 ----------------------------------------------------- N2(BR) ----------------------------------------------------- 1181

1182 TVDITNLPAGDYTVRVSATDSGATTPVVATYPLRVNVDTTPPTVANAKSDIFVFKGVAIDTDVTATGNQPLKWATVTDDIAVTDIIGKNNLVGLTLDLNGNVTGTSNSGAGFYS 1295

1296 ----------------------------------------------------- N2(BR) ----------------------------------------------------- 1512

1513 ------------------------------------------------- SRR-2 / LPNTG -------------------------------------------------- 2273

**(viii)** *Strep. suis* 92-4172 Fap1 with X1 partial Big_3_4 pfam13754 (WP_024401386.1):

257 -------------------------------- N2(BR) --------------------------------- 556

557 VSGTAEAGTTLTVSFTTNGVITTATTTVGADGTWSVPVPSGTVLTTSTVVSAKTVDASDKTVDNSSAVSTATV 629

630 -------------------------------- N2(BR) --------------------------------- 812

813 ----------------------------- SRR-2 / LPNTG ----------------------------- 2510

**Figure S2 A and B.** Additional binding motifs in SRRPs. **(A)** Organisation of MucBP repeat motifs (pfam06458) in SrpA, pseudo-SrpA and SRRPs from *Strep. salivarius* and *Strep. parasanguinis* strains (N2(BR) and SRR-2 domains not drawn to scale). ^a^ Gene incorrectly annotated in genome; full-length SrpA translated in one reading frame; ^b^ to give the protein sequence indicated, the internal pseudogene fragment HSISS4_01308 must be translated in the opposite reading frame to the two adjoining fragments either due to mis-assembly of the genome or a gene inversion in the MucBP repeats (indicated by different shading of the relevant MucBP repeats); **(B)** occurrence of bacterial group 3 Ig-like domains (Big_3_5 superfamily) in the N2(BR) domains of SssP1 and other SRRPs from *Strep. suis* strains (N2(BR), SRR-2, N3 and SRR-3 domains not drawn to scale).
