# Supplementary material for: Serine-rich repeat proteins from gut microbes
Source: Gut Microbes. 2019 Apr 29;11(1):102–17. doi: 10.1080/19490976.2019.1602428 (PMC6973325; doi:10.1080/19490976.2019.1602428)
Supplement: Supplemental Material [file kgmi-11-01-1602428-s001.zip › Supplementary information/new_new_new_Figure S1A-C.docx]

**(A)**

# Program: needle (Needleman-Wunsch algorithm)

# Matrix: EBLOSUM62

# Gap_penalty: 10.0

# Extend_penalty: 0.5

#

# Length: 282

# Identity: 131/282 (**46.5%**)

# Similarity: 169/282 (**59.9%**)

# Gaps: 39/282 (13.8%)

# Score: 667.0

JIM8777 SrpB-BR 1 QEDEKTVLDQNVSEAELLVNIAKNYQAKLTDTAAKAEIQTAITTVQEEVT 50

:..:..|..|:..|| .|:|:..

PsrP-BR 1 --------------------VVGSQTAAATEATAK--------KVEEDRK 22

JIM8777 SrpB-BR 51 KSTTLIAASATNA---AYAEQRERLGNAVDNMMTKLTNAGFNGNSTVNGT 97

|..:...||.||. :||::|:|..::::.::..:..|.|:||:.|||.

PsrP-BR 23 KPASDYVASVTNVNLQSYAKRRKRSVDSIEQLLASINAAVFSGNTIVNGA 72

JIM8777 SrpB-BR 98 PAITSNLNLATGETKVYTGTGTDTNYNVPIYYTLKVTNDGSNLNFVYTVT 147

|||.::||:|..|||:|||||.|:.||:||||.|.||||||.|.|.||||

PsrP-BR 73 PAINASLNIAKSETKIYTGTGRDSFYNIPIYYKLTVTNDGSKLTFTYTVT 122

JIM8777 SrpB-BR 148 YVNPATSTLGNISRMSPGYSIYNTGTTNQTMFALGTGLKAPTTVTSYITN 197

||:|.|.||||:||||.|||||||||..|||..||.|||.|::|.:|||:

PsrP-BR 123 YVDPITKTLGNLSRMSRGYSIYNTGTIYQTMLTLGKGLKTPSSVKNYITD 172

JIM8777 SrpB-BR 198 SDGTQRSTPSPNPTPVTSDSSGYSWGQGFQMNGFQAKNSYGLTSTWTAKI 247

.||.| ....|.:.:|:..|||:||.|.||||..||..|||||:||..|

PsrP-BR 173 KDGVQ--VQYYNMSTMTTQGSGYTWGNGAQMNGLFAKRGYGLTSSWTVPI 220

JIM8777 SrpB-BR 248 IGDDTSFTFSPYAGKTDNTSVNFFNG------ 273

.|.||||||:|||.|||....|:||.

PsrP-BR 221 TGTDTSFTFTPYAAKTDKIRTNYFNSKGKIVE 252

**(B)**

# Program: needle

# Matrix: EBLOSUM62

# Gap_penalty: 10.0

# Extend_penalty: 0.5

#

# Length: 71

# Identity: 37/71 (**52.1%**)

# Similarity: 46/71 (**64.8%**)

# Gaps: 2/71 ( 2.8%)

# Score: 189.5

SrpB-BR 390 QTMFALGTGLKAPTTVTSYITNSDGTQRSTPSPNPTPVTSDSSGYSWGQGFQMNGFQAKNSYGLTSTWTAK 460

|||..||.|||.|::|.:|||:.||.| ....|.:.:|:..|||:||.|.||||..||..|||||:||..

PsrP-BR 273 QTMLTLGKGLKTPSSVKNYITDKDGVQ--VQYYNMSTMTTQGSGYTWGNGAQMNGLFAKRGYGLTSSWTVP 341

**(C)**


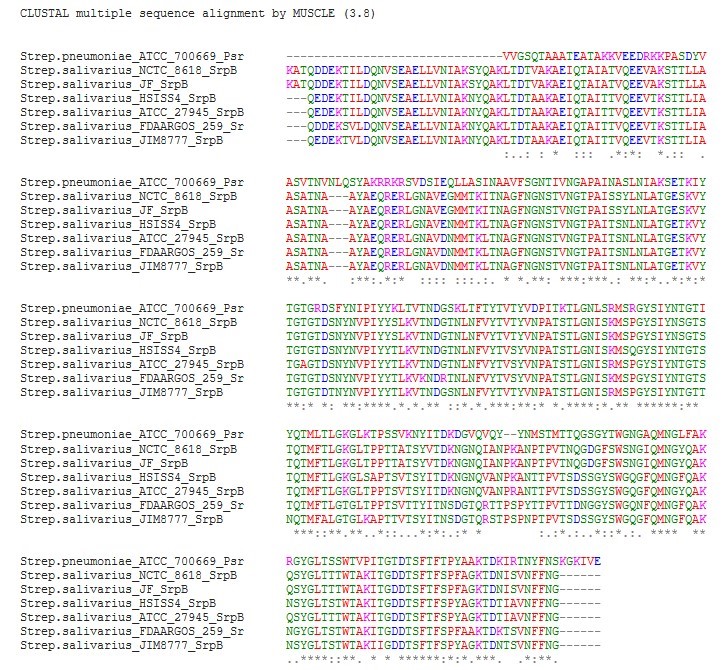


**Figure S1 A-C.** Alignments of *Strep. salivarius* SrpB-BRs with *Strep. pneumoniae* ATCC 700669 PsrP-BR. **(A)** Pairwise global alignment of *Strep. salivarius* JIM8777 SrpB-BR (aa positions 215-487 in the precursor sequence) with *Strep. pneumoniae* ATCC 700669 PsrP-BR (aa positions 123-374 in the precursor sequence) using the Needleman-Wunsch algorithm (EBI Tools: https://www.ebi.ac.uk/Tools/psa/emboss_needle/). The keratin 10 (KRT10) binding region of PsrP is highlighted in yellow, **(B)** pairwise global alignment of *Strep. salivarius* JIM8777 SrpB-BR over the 71 aa PsrP KRT10-binding region, **(C)** MUSCLE^33^ multiple sequence alignment in ClustalW2 format of PsrP-BR with six *Strep. salivarius* SrpB-BRs with the 71 aa PsrP KRT10-binding region highlighted in the yellow box.
