# Supplementary material for: Serine-rich repeat proteins from gut microbes
Source: Gut Microbes. 2019 Apr 29;11(1):102–17. doi: 10.1080/19490976.2019.1602428 (PMC6973325; doi:10.1080/19490976.2019.1602428)
Supplement: Supplemental Material [file kgmi-11-01-1602428-s001.zip › Supplementary information/new_new_new_Table S3A&B.pdf]

Table S3A and B. Pairwise global alignments of selected BR sequences using the Needleman-Wunsch algorithm (EBI Tools: https://www.ebi.ac.uk/Tools/psa/emboss_needle/)
Amino acid % identity **(A)** and % similarity **(B)** values are colour-coded as follows: >90% pink; >80-90% orange; >70-80% light blue; >60-70% dark blue; >50-60% purple; >40-50% lime; >30-40% grey; >20-30% yellow; 0-20% unshaded.

(A) aa % Identity

SRRP-BR	*L. reuteri* ATCC 53608 SRRP 1	*L. reuteri* 100-23 SRRP 2	*L. reuteri* ATCC 53608 pseudo-SRRP 3	*L. reuteri* 100-23 pseudo-SRRP 4	*Strep. salivarius* JIM8777 SrpA 5	*Strep. salivarius* JIM8777 SrpB 6	*Strep. salivarius* JIM8777 SrpC 7	*Strep. parasanguinis* FW213 Fap1 8	*Strep. gordonii* M99 GspB 9	*Strep. gordonii* DL1 Hsa 10	*Strep. pneumoniae* ATCC 700669 PsrP 11	*Staph.haemolyticus* JCSC1435 SraP 12	*Staph. aureus* N315 SraP 13	*Strep. sanguinis* SK36 SrpA 14	*Strep. agalactiae* NEM316 Srr-1 15	*Strep. agalactiae* J48 Srr-2 16
**1** *L. reuteri* ATCC 53608 SRRP	100.0	44.8	10.9	25.5	16.0	13.5	14.9	13.0	16.8	6.9	13.0	13.1	9.7	10.8	16.7	10.8
**2** *L. reuteri* 100-23 SRRP		100.0	10.4	26.2	11.8	15.2	16.4	18.2	19.5	11.4	10.8	10.7	14.0	10.0	16.2	13.6
**3** *L. reuteri* ATCC 53608 pseudo-SRRP			100.0	10.0	11.3	23.9	10.3	19.0	15.3	20.7	25.3	30.7	11.2	12.9	12.6	17.3
**4** *L. reuteri* 100-23 pseudo-SRRP				100.0	11.2	13.5	15.6	15.1	13.8	9.0	11.3	8.2	17.7	8.7	18.7	13.0
**5** *Strep. salivarius* JIM8777 SrpA					100.0	23.9	17.2	14.0	11.7	9.7	13.3	10.4	19.3	8.1	17.4	10.9
**6** *Strep. salivarius* JIM8777 SrpB						100.0	15.6	22.5	14.1	17.0	46.5	35.2	12.0	15.2	15.8	10.4
**7** *Strep. salivarius* JIM8777 SrpC							100.0	14.4	13.4	10.9	9.4	10.4	17.7	11.0	15.6	10.0
**8** *Strep. parasanguinis* FW213 Fap1								100.0	13.4	13.6	17.5	13.7	9.2	13.6	15.7	18.5
**9** *Strep. gordonii* M99 GspB									100.0	19.9	16.4	10.8	18.4	18.3	9.5	17.1
**10** *Strep. gordonii* DL1 Hsa										100.0	11.2	6.5	5.9	50.0	5.2	12.4
**11** *Strep. pneumoniae* ATCC 700669 PsrP											100.0	38.8	12.9	7.5	7.6	13.2
**12** *Staph.haemolyticus* JCSC1435 SraP												100.0	12.5	6.4	9.7	10.8
**13** *Staph. aureus* N315 SraP													100.0	9.3	12.9	13.7
**14** *Strep. sanguinis* SK36 SrpA														100.0	11.7	14.4
**15** *Strep. agalactiae* NEM316 Srr-1															100.0	27.6
**16** *Strep. agalactiae* J48 Srr-2																100.0

(B) aa % Similarity

SRRP-BR	*L. reuteri* ATCC 53608 SRRP 1	*L. reuteri* 100-23 SRRP 2	*L. reuteri* ATCC 53608 pseudo-SRRP 3	*L. reuteri* 100-23 pseudo-SRRP 4	*Strep. salivarius* JIM8777 SrpA 5	*Strep. salivarius* JIM8777 SrpB 6	*Strep. salivarius* JIM8777 SrpC 7	*Strep. parasanguinis* FW213 Fap1 8	*Strep. gordonii* M99 GspB 9	*Strep. gordonii* DL1 Hsa 10	*Strep. pneumoniae* ATCC 700669 PsrP 11	*Staph.haemolyticus* JCSC1435 SraP 12	*Staph. aureus* N315 SraP 13	*Strep. sanguinis* SK36 SrpA 14	*Strep. agalactiae* NEM316 Srr-1 15	*Strep. agalactiae* J48 Srr-2 16
**1** *L. reuteri* ATCC 53608 SRRP	100.0	61.5	19.6	36.9	27.4	22.0	26.7	25.0	27.5	12.5	20.0	21.2	16.9	16.5	30.3	18.5
**2** *L. reuteri* 100-23 SRRP		100.0	16.2	39.2	18.6	25.4	26.9	28.4	30.5	18.8	15.8	14.4	25.2	16.5	29.7	21.9
**3** *L. reuteri* ATCC 53608 pseudo-SRRP			100.0	18.2	17.9	34.1	16.1	28.6	23.3	29.7	38.6	44.5	18.5	21.2	20.1	30.5
**4** *L. reuteri* 100-23 pseudo-SRRP				100.0	16.4	19.3	26.9	24.3	22.5	13.9	17.0	12.7	29.3	14.3	35.4	20.1
**5** *Strep. salivarius* JIM8777 SrpA					100.0	28.9	27.8	23.8	21.2	16.4	21.6	16.5	32.4	15.5	29.7	20.3
**6** *Strep. salivarius* JIM8777 SrpB						100.0	22.2	33.0	22.9	27.1	59.9	42.5	18.1	24.1	26.6	17.0
**7** *Strep. salivarius* JIM8777 SrpC							100.0	22.2	21.9	16.9	15.8	13.5	28.3	17.3	25.7	15.6
**8** *Strep. parasanguinis* FW213 Fap1								100.0	22.8	25.1	26.6	21.2	17.8	22.1	24.8	29.8
**9** *Strep. gordonii* M99 GspB									100.0	30.2	24.8	17.4	28.6	27.0	15.6	29.8
**10** *Strep. gordonii* DL1 Hsa										100.0	20.3	9.3	7.8	62.7	9.9	25.1
**11** *Strep. pneumoniae* ATCC 700669 PsrP											100.0	46.5	19.0	13.3	14.3	21.1
**12** *Staph.haemolyticus* JCSC1435 SraP												100.0	17.0	10.0	17.5	15.6
**13** *Staph. aureus* N315 SraP													100.0	13.7	24.5	21.7
**14** *Strep. sanguinis* SK36 SrpA														100.0	20.1	24.5
**15** *Strep. agalactiae* NEM316 Srr-1															100.0	45.4
**16** *Strep. agalactiae* J48 Srr-2																100.0
