# Supplementary material for: Serine-rich repeat proteins from gut microbes
Source: Gut Microbes. 2019 Apr 29;11(1):102–17. doi: 10.1080/19490976.2019.1602428 (PMC6973325; doi:10.1080/19490976.2019.1602428)
Supplement: Supplemental Material [file kgmi-11-01-1602428-s001.zip › Supplementary information/new_Table S1.pdf]

Table S1. Strains of commensal species with a SecA2-SecY2 accessory secretion system and SRRP adhesin(s)[a]

Species	Strain	Genome accession number	SRRP gene ID(s) or locus tag(s)	pseudo-SRRP gene ID(s) or locus tag(s)
L. reuteri	ATCC 53608	LN906634-906636	LRATCC53608_0906	LRATCC53608_0916-_0917
	100-23	IMG: 2500069000	2500070902 (“Lr_70902”)	2500070903-2500070904 (“Lr_70903-_70904”)
	TD1	CP006603	N134_05915	N134_05970-_05965
	I49	NZ_CP015408	A4V07_RS03935-_RS03925[b]	—
	1366	NBBG01000001-01000093	B6J74_RS03710	—
	LTH5448	NZ_JOOG01000001-01000036	HN00_RS06750[c]	HN00_RS06765-_RS09675[c]
	121	MKQH01000001-01000014	BJI45_RS02085[b]	BJI45_RS02135-_RS02140
	ZLR003	NZ_CP014786	ADV92_RS10755[b]	ADV92_RS10805-_RS10810
	KLR1001	MIME01000001-01000145	BHL74_RS10285[b]	BHL74_RS10235-_RS10230
	KLR1002	MIMF01000001-01000392	BHL85_RS12575[b]	BHL85_RS12625-_RS12630
	KLR1004	MIMH01000001-01000154	BHL89_RS06905-_RS06910[b]	BHL89_RS06855[d]
	KLR3004	MIMT01000001-01000149	BHL81_RS01145[b]	BHL81_RS01195-_RS01200
	KLR4001	MIMW01000001-01000136	BHL84_RS04875	BHL84_RS04815
	3c6	LN887305-887505	LR3C6_01537A-_01537	LR3C6_00253A-_00253
	20-2	LN887506-887693	LR202_00269A-_00269	LR202_00347A-_00347
	lp167-67	LN887694-887827	LRLP167_00243A-_00243	LRLP167_00253-_00254
	pg-3b	LN887201-887304	LRPG3B_00922A-_00922	LRPG3B_00234A-_00234
	lpuph	NZ_AEAX01000001-01000127	—[d]	ECQ_RS11170[d] & ECQ_RS0107090-_RS0107085
	mlc3	NZ_AEAW01000001-01000126	—[d]	ECM_RS10935[d,e]
	480_44	MBLQ01000001-01000154	—[d]	BBP10_RS03300[d]
	482_46	MBLR01000001-01000188	—[d]	BBP11_RS07570[d]
	482_54	MBLS01000001-01000186	—[d]	BBP12_RS00010[d]
	484_32	MBLT01000001-01000523	—[d]	BBP13_RS14455[d]
	484_39	MBLU01000001-01000191	—[d]	BBP14_RS11505[d]
	KLR2001	MIMI01000001-01000149	—[d]	BHL90_RS06020[d] & BHL90_RS06080-_ RS06085
	KLR2002	MIMJ01000001-01000169	—[d]	BHL91_RS06735[d] & BHL91_RS06785-_RS06790
	KLR2003	MIMK01000001-01000149	—[d]	BHL92_RS06520[d] & BHL92_RS06570-_RS06575
	KLR2004	MIML01000001-01000140	—[d]	BHL93_RS00715-_RS00720[d] & BHL93_RS00770-_RS00775
	KLR2007	MIMO01000001-01000136	—[d]	BHL76_RS05680-_RS05685[d] & BHL76_RS05735-_RS05740
	KLR2008	MIMP01000001-01000143	—[d]	BHL77_RS05585[d] &

				BHL77_RS05535-_RS05530
	KLR3002	MIMR01000001-01000228	–[d]	BHL79_RS06455-_RS06450[d] & BHL79_RS06400-_RS06395
	KLR3003[f]	MIMS01000001-01000142	–	BHL80_RS07775-_RS07780
	KLR3005	MIMU01000001-01000172	–[d]	BHL82_RS05895[d] & BHL82_RS05955[d]
	KLR3006	MIMV01000001-01000257	–[d]	BHL83_RS05150-_RS05145[d] & BHL83_RS05095-_RS05090
	CECT8605	MWVS01000001-01000207	–[d]	B5D07_RS10730-_RS10735[d] & B5D07_RS10785-_RS10790
	I5007	NC_021494-021504	–	LRI_RS04175-_RS04185 & LRI_RS04235-_RS04240
	LR0	MWIJ01000001-01000075	–	B2G46_RS05900-_RS05885 & B2G46_RS05955-_RS05950
	TMW1.112	NZ_JOKX02000001-02000012	–	HF82_RS02820-_RS02815
	TMW1.656	IMG: 2534682350	–	LR4_00083[g] & LR4_00669-_00668
	LTH2584	NZ_JOSX01000001-01000025	–	LR3_RS06575-_RS10625 & LR3_RS10630-_RS06580
L. oris	F0423	AFTL01000001-01000020	HMPREF9102_0778 & HMPREF9102_0779	–
	PB013-T2-3	AEKL01000001-01000089	HMPREF9265_0662	HMPREF9265_0661
L. salivarius	NIAS840	NZ_AFMN01000001-01000004	NIAS840_RS03930	NIAS840_RS00005[h] & NIAS840_RS10015
	JCM1046	NZ_CP007646-007650	LSJ_RS11575[i]	LSJ_RS00310-_RS11750 & LSJ_RS11780[i]
	L12	IMG: 2540341228	–	L120_00174 & L120_00797
	cp400	NZ_CBVR010000001-010000089	–	LSCP400_RS00005-_RS00010 & LSCP400_RS04945
	SMXD51	NZ_AICL01000001-01000010	–	SMXD51_RS09695-_RS09710 & SMXD51_RS00010[j]
L. johnsonii	NCC 533	AE017198	LJ_0391 & LJ_1711[k]	–
	N6.2	CP006811	T285_07275[l]	T285_01855-_01860
	DPC6026	NC_017477	–	LJP_RS01910-_RS09490 & LJP_RS09630-_RS09450[m]
	L6	IMG: 2529292694	–	L60_01856-_01857
	16	LIGY01000001-01000156	–	LJ16_RS02840
	W1	LSNG01000001-01000049	–	AYJ53_RS01730

L. fructivorans	DmCS_002	JOJZ01000001-01000025	LfDm3_0405	–
L. gasseri	987_LJOH[n]	JUKW01000001-01000081	–	ADF22_RS09755-_RS09750
	JV-V03	GL379580-379587	–	HMPREF0514_10057-_10058
	K7	KL402718-402725	–	LK7_01678
	L3	IMG: 2518645515	–	LGS03_00064-_00066
L. mucosae	LM1[o]	NZ_CP011013	–	LBLM1_RS11745, LBLM1_RS03980[p] & LBLM1_ RS11555[p]
	DPC 6426	JSWI01000001-01000072	–	OC62_RS10615
L. murinus	ASF361	KB822402-822412	–	ASF361_01512
L. rhamnosus	L33	IMG: 2518645521	–	LRH33_01715-_01714
L. nagelii	DSM 13675	AZEV01000001-01000044	–	FD45_GL000390[d,q]
Lc. lactis subsp. *cremoris*	KW2	CP004884	kw2_0799	–
Strep. salivarius[r]	JIM8777	FR873482	SALIVA_1456, SALIVA_1457 & SALIVA_1458	–
	JF	CP014144	AWB63_06780, AWB63_06785 & AWB63_06790	–
	NCTC 8618	NZ_CP009913	SSAL8618_RS07150, SSAL8618_RS07155 & SSAL8618_RS10410	–
	HSISS4	CP013216	HSISS4_01305 & HSISS4_01306	HSISS4_01309-_01307
	ATCC 27945	NZ_CP015282	NX99_RS00100 & NX99_RS00105	NX99_RS00110
	ATCC 25975	NZ_CP015283	V471_RS05855	V471_RS05860-_RS05865 & V471_RS05870-_RS05875
	K12	ALIF01000001-01000007	RSSL_00002	RSSL_00001[d]
	57.I	CP002888-002889	–	Ssal_00697-_00700, Ssal_00701-_00703 & Ssal_00708-_00710
Strep. vestibularis[r]	F0396	AEKO01000001-01000011	HMPREF9192_1022	–

Strep. mitis[s]	B6	FN568063	smi_1662	–
	ATCC 6249	NZ_GL397179-397186	–	HMPREF8571_RS10990
Strep. oralis[s]	Uo5	FR720602	SOR_1583	–
Strep. cristatus[s]	AS 1.3089	NC_021175	–	I872_RS07245 & I872_RS06615-_RS10965[t]
	CC5A	NZ_JYGJ01000001-01000007	TW70_RS03985(-TW70_01362)[u]	TW70_RS05530
	ATCC 51100	NZ_AFUE01000001-01000010	HMPREF9960_RS10050-_RS10040[b,v,w] & HMPREF9960_RS10120-_RS10125[b,w]	HMPREF9960_RS08055
Strep. thoraltensis	DSM 12221	NZ_KB904586-904633	–	A3IA_RS11895-_RS12265
Strep. sp. DD12	DD12[o]	NZ_KQ969495-969505	STRDD12_RS03215	STRDD12_RS02690, STRDD12_RS03200-_RS03195 & STRDD12_RS03210

[a] Strains that possess a SecA2-SecY2 cluster but lack a SRRP (linked or unlinked to the cluster) have been excluded

[b] Gene incorrectly annotated in genome; full-length SRRP translated in one ORF

[c] Both genes are unlinked to the LTH5448 SecA2-SecY2 cluster (HN00_RS05550-_RS05600)

[d] Possible intact SRRP with partial gene sequence at the end of a draft genome contig, and in the cases of strains KLR2004, KLR2007, KLR3002, KLR3006 and CECT8605, annotated incorrectly as two pseudogene fragments

[e] Unlinked to the mlc3 SecA2-SecY2 cluster (ECM_RS0104210-_RS0104260)

[f] Strain KLR3003 has a truncated SecA2-SecY2 cluster with only genes encoding SecA2, GtfA, GtfB, a small hypothetical protein and a pseudo-SRRP

[g] Unlinked to the TMW1.656 SecA2-SecY2 cluster (LR4_00668-_00683)

[h] Unlinked to the SecA2-SecY2 cluster which is split into two regions of the draft genome (NIAS840_RS03905-_RS03930 and NIAS840_RS01240-_RS10015)

[i] SRRP (LSJ_RS11575) and pseudo-SRRP (LSJ_RS11780) are unlinked to the JCM1046 SecA2-SecY2 cluster (LSJ_RS00285-_RS00380)

[j] The SecA2-SecY2 cluster is found on two adjacent contigs in the SMXD51 draft genome (SMXD51_RS00595-_RS09705 and SMXD51_RS09710-_RS00700) but pseudogene SMXD51_RS00010 is unlinked to these

[k] Unlinked to the NCC 533 SecA2-SecY2 cluster (LJ_0384-_0393)

[l] Unlinked to the N6.2 SecA2-SecY2 cluster (T285_01815-_01870) but is linked to four other Gtf genes (T285_07255-_07275)

[m] Unlinked to the DPC 6026 SecA2-SecY2 cluster (LJP_RS01870-_RS01945) but is linked to two other Gtf genes (LJP_RS07505-_RS07510)

[n] Although included in this table, strain 987_LJOH is a clinical isolate from the wound of an intensive care unit patient, originally identified as *L. johnsonii*

[o] Strains with two SecA2-SecY2 clusters but with one cluster lacking Gtf genes: *L. mucosae* LM1 (LBLM1_ RS03980-_RS04070 & LBLM1_ RS11555-_ RS04660) and *Strep*. sp. DD12 (STRDD12_RS02690-_RS02715 & STRDD12_RS03195-_RS03255), each with their own SRRP and/or pseudo-SRRPs

[p] Pseudogene fragments translated in forward frames (not the reverse complement as indicated in the published genome)

[q] Translated in the opposite reading frame to that annotated in the genome from nt 112572–115183

[r] Belonging to the salivarius group of viridans group streptococci (VGS) that are usually of low virulence but some strains can be a major cause of sepsis, pneumonia, meningitis and subacute endocarditis; strains listed here are classed as commensal, probiotic or lantibiotic

[s] Belonging to the mitis group of VGS that includes pathogens *Strep. pneumoniae*, *Strep. gordonii*, *Strep. sanguinis* and *Strep. parasanguinis*, causing pneumonia, sepsis and organ failure, but strains listed here are classed as commensal

[t] Unlinked to the AS 1.3089 SecA2-SecY2 cluster (I872_RS07225-_RS07275)

[u] Formerly annotated as full-length SrpA ORF9R in GenBank accession number U96166 (locus SCU96166), unlinked to the CC5A SecA2-SecY2 cluster (TW70_RS05500-_RS05550) but linked to a GtfB gene

[v] Formerly annotated as complete SraP HMPREF9960_1919 in the original genome assembly ASM22276.1

[w] Both SRRP genes unlinked to the ATCC 51100 SecA2-SecY2 cluster (HMPREF9960_RS08025-_RS08075) although HMPREF9960_RS10050-_RS10040 is linked to a GtfB gene
