# Supplementary material for: Serine-rich repeat proteins from gut microbes
Source: Gut Microbes. 2019 Apr 29;11(1):102–17. doi: 10.1080/19490976.2019.1602428 (PMC6973325; doi:10.1080/19490976.2019.1602428)
Supplement: Supplemental Material [file kgmi-11-01-1602428-s001.zip › Supplementary information/new_Table S2.pdf]

Table S2. Domain organisation of SRRPs including composition of serine-rich repeats (SRRs) in each SRR-2 domain

Bacterial strain	SRRP[a]	Gene or locus tag(s)	Precursor size (aa)	Sec. signal (aa)	AST domain (aa)	SRR-1 domain (aa)	BR domain (aa)	SRR-2 domain			
								Length (aa)	Total no. of each SRR type	SRR consensus sequence(s)	No. of most frequent SRR(s)
**Commensals/Non-pathogens**											
L. reuteri ATCC 53608	SRRP$_{53608}$	LRATCC53608_0906	1125	88	7	127	446	414	18<br>7	SLSNSVSMSE<br>STSASVSASE	11<br>4
L. reuteri ATCC 53608	SRRP$_{53608p}$	(LRATCC53608_0916)-<br>LRATCC53608_0917[b]	795[b]	(91)	(7)	(85) +<br>65	216	258	9	STSASVSASE	2
L. reuteri 100-23	SRRP$_{100\text{-}23}$	Lr_70902	2180	91	8	102	486<br>[551][c]	1418<br>[1353][c]	103<br>7	SLSNSVSMSE<br>STSASVSASE	91<br>1
L. reuteri 100-23	SRRP$_{100\text{-}23p}$	Lr_70903-(Lr_70904)[b,d]	2626[b]	90	8	103	449	1669 +<br>(231)	155<br>12	SLSNSVSMSE<br>SLSESRSGSA	91<br>3
L. reuteri 480_44	SRRP$_{48044p}$	BBP10_RS03300[e]	870[d]	91	8	100	426	245	21	SLSNSVSMSE	19
L. reuteri 482_46	SRRP$_{48246p}$	BBP11_RS07570[e]	868[d]	91	8	100	426	243	21	SLSNSVSMSE	19
L. reuteri 482_54	SRRP$_{48254p}$	BBP12_RS00010[e]	876[d]	91	8	100	426	251	22	SLSNSVSMSE	20
L. reuteri TMW1.112	SRRP$_{1.112p}$	HF82_RS02820-<br>(HF82_RS02815)[b,d]	1402[b]	91	7	128	493	306 +<br>(377)	63	SLSNSVSMSE	56
L. reuteri LTH5448	SRRP$_{5488}$	HN00_RS06750	1314	90	8	101	457	582	42	SLSNSVSMSE	26
L. reuteri LTH5448	SRRP$_{5488p}$	HN00_RS06765-<br>(HN00_RS09675)[b]	1339	(91)	(8)	(54) +<br>47	538	174 +<br>(352)	28	SLSNSVSMSE	21
L. reuteri TMW1.656	SRRP$_{1.656\text{-}1p}$	LR4_00083[d]	1074	90	8	101	458	389	25	SLSNSVSMSE	11
L. reuteri TMW1.656	SRRP$_{1.656\text{-}2p}$	(LR4_00669)-<br>LR4_00668[b]	950	(78) +<br>13	8	117	525	209	10	SNSVSASE	8
L. reuteri LTH2584	SRRP$_{2584\text{-}1p}$	LR3_RS06575-<br>(LR3_RS10625)[b,d]	1860[b]	90	8	101	458	174 +<br>(952)	92	SLSNSVSMSE	60
L. reuteri LTH2584	SRRP$_{2584\text{-}2p}$	LR3_RS10630-<br>(LR3_RS06640-<br>_RS06580)[b,d]	2273[b]	93	8	99	536	176 +<br>(1299)	156	SNSVSASE	148
L. reuteri 484_39	SRRP$_{48439p}$	BBP14_RS11505[e]	1529[d]	91	7	100	450	881	83	SLSQSESAST	61
L. reuteri LR0	SRRP$_{LR0\text{-}1p}$	(B2G46_RS05955)-<br>B2G46_RS05950[b]	979[b]	(88)	(7)	(74) +<br>53	439	245	9	SLSQSESAST	3

L. reuteri LR0	SRRP$_{LR0-2p}$	B2G46_RS05900-(B2G46_RS05890-RS05885)[b,d]	2038[b]	91	7	100	450	794 + (520)	114	SLSQSESAST	103
L. reuteri TD1	SRRP$_{TD1}$	N134_05915	3138	91	7	100	450	2414	223<br>3	SLSQSESAST<br>SASTSASTSASL	202<br>1
L. reuteri TD1	SRRP$_{TD1p}$	(N134_05970)-N134_05965[b]	969[b]	(88)	(7)	(74) + 53	439	235	8	SLSQSESAST	2
L. reuteri I49	SRRP$_{I49}$	A4V07_RS03935-_RS03925[f]	3230	91	7	100	449	2507	231<br>3	SLSQSESAST<br>SASTSASTSAST	155<br>1
L. reuteri lpuph	SRRP$_{lpuph-1p}$	(ECQ_RS0107090)-ECQ_RS0107085[b]	943[b]	(88)	(7)	(74) + 53	439	239	9	SLSQSESAST	3
L. reuteri lpuph	SRRP$_{lpuph-2p}$	ECQ_RS11170[e]	745	91	7	100	450	97	6	SLSQSESAST	5
L. reuteri 1366	SRRP$_{1366}$	B6J74_RS03710	1497	91	7	88	464	771	60	SLSQSESAST	34
L. reuteri 20-2	SRRP$_{20-2}$	LR202_00269A-_00269	1240	88	7	127	457	487	30	SLSQSESAST	11
L. reuteri pg-3b	SRRP$_{pg-3b}$	LRPG3B_00922A-_00922	1089	88	7	127	446	347	16	SLSQSESAST	6
L. reuteri pg-3b	SRRP$_{pg-3bp}$	LRPG3B_00234A-(LRPG3B_00234)[b,d]	1067[b]	91	7	88	469	162 + (174)	19	SLSNSVSMSE	12
L. reuteri 3c6	SRRP$_{3c6}$	LR3C6_01537A-_01537	1050	88	7	127	457	297	10	SLSQSESAST	4
L. reuteri 3c6	SRRP$_{3c6p}$	LR3C6_00253A-(LR3C6_00253)[b,d]	1086[b]	91	7	118	230	276 + (289)	29	STSASVSASE	18
L. reuteri lp167-67	SRRP$_{lp167}$	LRLP167_00243A-_00243	1230	88	7	127	451	483	20<br>14	STSASVSASE<br>SLSNSVSMSE	11<br>6
L. reuteri lp167-67	SRRP$_{lp167p}$	(LRLP167_00253)-LRLP167_00254[b]	785[b]	(91)	(7)	(85) + 65	230	232	8	STSASVSASE	1
L. reuteri I5007	SRRP$_{I5007-1p}$	LRI_RS04175-(LRI_RS04180-_RS04185)[b,d]	1327[b]	88	7	127	446	281 + (304)	31<br>15	STSASVSASE<br>SLSNSVSMSE	18<br>13
L. reuteri I5007	SRRP$_{I5007-2p}$	(LRI_RS04235)-LRI_RS04240[b]	795[b]	(91)	(7)	(84) + 66	230	242	9	STSASVSASE	2
L. reuteri 121	SRRP$_{121}$	BJI45_RS02085[f]	1785	88	7	127	446	1043	73<br>15	STSASVSASE<br>SLSNSVSMSE	47<br>10
L. reuteri 121	SRRP$_{121p}$	(BJI45_RS02135)-BJI45_RS02140[b]	805[b]	(91)	(7)	(84) + 66	230	252	10	STSASVSASE	2
L. reuteri ZLR003	SRRP$_{ZLR3}$	ADV92_RS10755[f]	1385	88	7	127	446	643	34<br>14	STSASVSASE<br>SLSNSVSMSE	21<br>10
L. reuteri ZLR003	SRRP$_{ZLR3p}$	(ADV92_RS10805)-ADV_RS10810[b]	795[b]	(91)	(7)	(84) + 66	230	242	9	STSASVSASE	2
L. reuteri KLR1001	SRRP$_{1001}$	BHL74_RS10285[f]	1273	88	7	127	446	531	24<br>13	STSASVSASE<br>SLSNSVSMSE	14<br>11

L. reuteri KLR1001	SRRP$_{1001p}$	(BHL74_RS10235)-<br>BHL74_RS10230[b]	815[b]	(91)	(7)	(84) +<br>66	230	262	11	STSASVSASE	4
L. reuteri KLR1002	SRRP$_{1002}$	BHL85_RS12575[f]	1275	88	7	127	446	533	26<br>11	STSASVSASE<br>SLSNSVSMSE	16<br>9
L. reuteri KLR1002	SRRP$_{1002p}$	(BHL85_RS12625)-<br>BHL85_RS12630[b]	795[b]	(91)	(7)	(84) +<br>66	230	242	9	STSASVSASE	2
L. reuteri KLR1004	SRRP$_{1004\text{-}2}$	BHL89_RS06905-<br>BHL89_RS06910[f]	782	91	7	137	230	242	9	STSASVSASE	2
L. reuteri KLR2001	SRRP$_{2001\text{-}2p}$	BHL90_RS06080-<br>_RS06085[d]	934	91	7	88	479	269	23	SLSNSVSMSE	22
L. reuteri KLR2002	SRRP$_{2002\text{-}2p}$	(BHL91_RS06785)-<br>BHL91_RS06790[b]	805[b]	(91)	(7)	(84) +<br>66	230	252	10	STSASVSASE	2
L. reuteri KLR2003	SRRP$_{2003\text{-}2p}$	(BHL92_RS06570)-<br>BHL92_RS06575[b]	805[b]	(91)	(7)	(84) +<br>66	230	252	10	STSASVSASE	2
L. reuteri KLR2004	SRRP$_{2004\text{-}2p}$	(BHL93_RS00770)-<br>BHL93_RS00775[b]	795[b]	(91)	(7)	(84) +<br>66	230	242	9	STSASVSASE	2
L. reuteri KLR2007	SRRP$_{2007\text{-}2p}$	(BHL76_RS05735)-<br>BHL76_RS05740[b]	805[b]	(91)	(7)	(84) +<br>66	230	252	10	STSASVSASE	2
L. reuteri KLR2008	SRRP$_{2008\text{-}2p}$	(BHL77_RS05535)-<br>BHL77_RS05530[b]	805[b]	(91)	(7)	(84) +<br>66	230	252	10	STSASVSASE	2
L. reuteri KLR3002	SRRP$_{3002\text{-}2p}$	(BHL79_RS06400)-<br>BHL79_RS06395[b]	755[b]	(91)	(7)	(84) +<br>66	230	202	7	STSASVSASE	1
L. reuteri KLR3003	SRRP$_{3003\text{-}2p}$	(BHL80_RS07775)-<br>BHL80_RS07780[b]	733[b]	(91)	(7)	(84) +<br>66	230	180	5	STSASVSASE	0
L. reuteri KLR3004	SRRP$_{3004}$	BHL81_RS01145[f]	1230	88	7	127	451	483	19<br>13	STSASVSASE<br>SLSNSVSMSE	9<br>10
L. reuteri KLR3004	SRRP$_{3004p}$	(BHL81_RS01195)-<br>BH81_RS01200[b]	805[b]	(91)	(7)	(84) +<br>66	230	252	10	STSASVSASE	2
L. reuteri KLR3005	SRRP$_{3005\text{-}2p}$	BHL82_RS05955[d]	1024	91	7	88	469	369	29	SLSNSVSMSE	28
L. reuteri KLR3006	SRRP$_{3006\text{-}2p}$	(BHL83_RS05095)-<br>BHL83_RS05090[b]	795[b]	(91)	(7)	(84) +<br>66	230	242	9	STSASVSASE	2
L. reuteri KLR4001	SRRP$_{4001}$	BHL84_RS04875	1286	91	8	102	539	471	10<br>7	SLSNSVSMSE<br>STSASLSASL	6<br>1
L. reuteri<br>CECT8605	SRRP$_{8605\text{-}2p}$	(B5D07_RS10785)-<br>B5D07_RS10790[b]	795[b]	(91)	(7)	(84) +<br>66	230	242	9	STSASVSASE	2
L. johnsonii N6.2	SRRP$_{N6.2}$	T285_07275	2537	62	3	155	1192[g]	1041	99	SLSNSVSMSE	81
L. johnsonii N6.2	SRRP$_{N6.2p}$	T285_01855-<br>(T285_01860)[b,d]	988[b]	60	63	143	490	102 +<br>(127)	17	SLSNSVSMSE	11
L. johnsonii NCC<br>533	SRRP$_{533\text{-}1}$	LJ_0391	1096	58	3	170	119	684	63	SLSNSVSMSE	46

L. johnsonii NCC 533	SRRP$_{533-2}$	LJ_1711	3039	62	3	155	1192[g]	1573	147	SLSNSVSMSE	109
L. johnsonii DPC6026	SRRP$_{6026-1p}$	LJP_RS01910-(LJP_RS09490)[b,d]	1149[b]	60	3	159	462	183 + (200)	36	SLSNSVSMSE	28
L. johnsonii DPC6026	SRRP$_{6026-2p}$	(LJP_RS09630-_RS07530)-LJP_RS09450[b]	2091[b]	(62)	(3)	(155)	(203) + 989[g]	625	53	SLSNSVSMSE	42
L. johnsonii W1	SRRP$_{W1p}$	AYJ53_RS01730[d]	817	58	3	153	481	122	10	SLSNSVSMSE	5
L. johnsonii 16	SRRP$_{16p}$	LJ16_RS02840[d]	724	60	3	153	480	28	-	-	-
L. fructivorans DmCS_002	SRRP$_{DmCS}$	LfDm3_0405	3377	55	1	229	612	2426	84<br>18<br>14<br>8	STSMSDSLSTSASDSA<br>SQSISTSNSGSASLSTSD<br>SVSGSASVSDSLSTSASGST<br>SDSLSNSTSK	14<br>8<br>4<br>2
L. mucosae LM1	SRRP$_{LM1p}$	LBLM1_RS11745[d]	4822	(53) + 42	13	114	230	4270	348	SASTSASLSASE	141
L. oris PB013-T2-3	SraP$_{T2-3p}$	HMPREF9265_0661	2934	(91) + 3	8	96	457	2230	121<br>4	SASTSASTSAST<br>MSSVSLATSTVR	7<br>3
L. oris PB013-T2-3	SRRP$_{T2-3}$	HMPREF9265_0662	1941	94	9	120	388	1272	93	SASTSASTSAST	9
L. oris F0423	SraP$_{F0423}$	HMPREF9102_0778	2428	94	8	96	457	1724	84<br>2	SASTSASTSAST<br>MSSVSLATSTVR	10<br>1
L. oris F0423	SRRP$_{F0423}$	HMPREF9102_0779	1238	94	9	120	388	568	35	SASTSASTSAST	5
L. salivarius NIAS840	SRRP$_{NIAS}$	NIAS840_RS03930	1035	101	19	84	434	329	22<br>1	SESLSTSTSSSL<br>SESLSTSSSL	17<br>1
L. salivarius NIAS840	SRRP$_{NIASp}$	NIAS840_RS00005[d]	986	101	19	99	455	312	24	SESLSTSTSSSL	18
L. salivarius JCM1046	SRRP$_{1046-2}$	LSJ_RS11575	900	101	19	85	432	161	11	SESLSTSSSL	7
L. salivarius SMXD51	SRRP$_{SMXD51-1p}$	SMXD51_RS09695-(SMXD51_RS09700-_RS09710)[b,d]	1233[b]	101	19	83	446	431 + (514)	69	SESLSTSTSSSL	59
L. salivarius SMXD51	SRRP$_{SMXD51-2p}$	SMXD51_RS00010[d]	969	101	19	99	458	292	21	SESLSTSTSSSL	13
L. nagelii DSM 13675	SRRP$_{13675p}$	FD45_GL000390[d,h]	870	97	23	150	358	242	16	SSSMSSSESASEST	7
Lc. lactis subsp. *cremoris* KW2	SRRP$_{KW2}$	kw2_0799	2338	97	13	39	134	2017	80<br>38	SLSESLTESLTEST<br>QDSKSESASLSDSLSDSA	69<br>7
Strep. salivarius JIM8777	SrpA[i]	SALIVA_1458	2312	90	32	93	572	563	8<br>6<br>27	SQSVSQSESEVVSK<br>SQSVSESEVVSK<br>SASASESASASE	4<br>3<br>8

Strep. salivarius JIM8777	SrpB	SALIVA_1457	2300	90	32	92	273	1765	55<br>2<br>53	SQSVSNSESASESASVSE<br>SnSSSLSnSnnnSn<br>SASASASASASA	13<br>-<br>4
Strep. salivarius JIM8777	SrpC (FliS)	SALIVA_1456	2835	90	32	132	607	1927	66<br>8<br>11<br>23	SQSVSNSESASESASVSE<br>SQSQSASTSASLSTVKSA<br>SVSSSLSESLKTSV<br>SASTSASASASV	21<br>2<br>4<br>1
Strep. salivarius JF	SrpA[i]	AWB63_06780	2213	90	32	93	568	381	11<br>7	SQSASLSTSASESASVSA<br>SASASESASASE	2<br>2
Strep. salivarius JF	SrpB	AWB63_06785	1988	90	31	90	276	1449	48<br>2<br>36	SQSVSNSESASESASVSE<br>SnSSSLSnSnnnSn<br>SASASASASASA	28<br>-<br>3
Strep. salivarius JF	SrpC (FliS)	AWB63_06790	2680	90	32	132	610	1769	43<br>10<br>12<br>15	SQSVSNSESASESASVSE<br>SQSQSASTSASLSTVKSA<br>SVSSSLSESLKTSV<br>SASASASASVSE	16<br>2<br>6<br>1
Strep. salivarius NCTC 8618	SrpA[i]	SSAL8618_RS10410	2019	90	32	93	568	381	11<br>7	SQSASLSTSASESASVSA<br>SASASESASASE	2<br>2
Strep. salivarius NCTC 8618	SrpB	SSAL8618_RS07155	1988	90	31	90	276	1449	48<br>2<br>36	SQSVSNSESASESASVSE<br>SnSSSLSnSnnnSn<br>SASASASASASA	28<br>-<br>3
Strep. salivarius NCTC 8618	SrpC (FliS)	SSAL8618_RS07150	2680	90	32	132	610	1769	43<br>10<br>12<br>15	SQSVSNSESASESASVSE<br>SQSQSASTSASLSTVKSA<br>SVSSSLSESLKTSV<br>SASASASASVSE	16<br>2<br>6<br>1
Strep. salivarius HSISS4	SrpA$_p$[i]	HSISS4_01309-(HSISS4_01308-_01307)[b,d]	1728[b]	90	32	93	575	269	8<br>7	SQSSSLSTSASESASVSA<br>SASTSASASASE	2<br>1
Strep. salivarius HSISS4	SrpB	HSISS4_01306	1134	90	32	92	273	597	14<br>2<br>16	SQSVSNSESASESASVSE<br>SnSSSLSnSnnnSn<br>SESASASASTSA	2<br>-<br>2
Strep. salivarius HSISS4	SrpC (FliS)	HSISS4_01305	1748	90	32	132	610	837	17<br>3<br>5<br>21	SQSVSNSESASESASVSE<br>SQSESASTSASLSTVKSA<br>SnSSSLSESLKTSn<br>SASASASTSASE	5<br>2<br>1<br>1
Strep. salivarius ATCC 27945	SrpA	NX99_RS00100	1983	90	32	95	572	527	8<br>6<br>21	SQSVSQSESEVVSK<br>SQSVSESEVVSK<br>SASASESASASE	4<br>3<br>5
Strep. salivarius ATCC 27945	SrpB	NX99_RS00105	1984	90	32	92	273	1445	45<br>3<br>33	SQSVSNSESASESASVSE<br>SnSSSLSnSnnnSA<br>SASTSASVSASE	25<br>1<br>8

Strep. salivarius ATCC 27945	SrpC$_{p}$ (FliS$_{p}$)	NX99_RS00110[b,d]	2764[b]	90	32	132	610	1071 + (782)	57<br>10<br>13<br>22	SQSVSNSESASESASVSE<br>SQSESASTSASLSTVKSA<br>SVSSSLSESLKTSL<br>SASTSASTSASE	12<br>2<br>2<br>3
Strep. salivarius ATCC 25975	SrpA$_{25975}$	V471_RS05855	2402	90	36	84	489	1654	99	SASESASTSASMSAST	60
Strep. salivarius ATCC 25975	SrpB$_{25975p}$	V471_RS05860-V471_RS05865[f]	4934	90	36	82	1442[g]	3226	194	SASESASTSASMSAST	145
Strep. salivarius ATCC 25975	SrpC$_{25975p}$	(V471_RS05870)-V471_RS05875[b]	1702[b]	(90)	(36)	(84)	(396) + 427[g]	620	33	SASESASTSASMSAST	17
Strep. salivarius 57.I	SrpA$_{57.Ip}$	Ssal_00697-(Ssal_00700)[b,d]	1909[b]	90	36	84	486	754 + (412)	68	SASESASTSASMSAST	41
Strep. salivarius 57.I	SrpB$_{57.Ip}$	(Ssal_00701)-Ssal_00703[b]	2081[b]	(90)	(36)	(104) + 4	1441[g]	340	18	SASESASTSASMSAST	9
Strep. salivarius 57.I	SrpC$_{57.Ip}$	Ssal_00708-(Ssal_00710)[b,d]	1919[b]	90	36	80	820[g]	572 + (272)	47	SASESASTSASMSAST	31
Strep. salivarius K12	SrpC (FliS)	RSSL_00002	2926	90	32	132	610	2015	71<br>6<br>10<br>3<br>20	SQSVSNSESASESASVSE<br>SQSQSASTSTSLSAVKSQ<br>SVSSSLSESnnnSV<br>SQSESASESnSVSE<br>SASTSASVSASE	6<br>2<br>3<br>1<br>2
Strep. vestibularis F0396	FliS	HMPREF9192_1022	2281	90	32	133	608	1366	62<br>2<br>11	SQSVSNSESASESASISE<br>SVSSSLSnSLKTSn<br>SASTSASESASE	5<br>-<br>3
Strep. mitis B6	MonX$_{B6}$	smi_1662	1591	92	25	101	728	605	20<br>15	SASQSASTSASKSAST<br>SASASASTSASESAST	18<br>13
Strep. oralis Uo5	MonX$_{Uo5}$	SOR_1583	1962	92	32	124	471	1199	98<br>38	SQSASVSS<br>SESASVSS	30<br>18
Strep. cristatus CC5A	SrpA	TW70_RS03985-(TW70_01362) [SCU96166_ORF9R][j]	3364	93	44	102	485	2577	22<br>35<br>86<br>53	SQSASASASNNQNSASISVSA<br>SASTSMSNSVSASISASV<br>SQSASASVSA<br>SQSASVSA	16<br>15<br>29<br>25
Strep. cristatus ATCC 51100	SraP	HMPREF9960_RS10050-_RS10040[f]	3058	93	44	102	485	2271	14<br>42<br>70<br>41	SQSASASASNNQNSASISVSA<br>SASTSMSNSVSASISASE<br>SQSASASVSA<br>SHSASVSA	6<br>13<br>27<br>8
Strep. cristatus ATCC 51100	SrpC (FliS)	HMPREF9960_RS10120-_RS10125[f]	4590	93	46	114	700	3574	35<br>161	SLSTSLSASISASDSAST<br>SQSVSTSASASASLSL	13<br>10
Strep. cristatus AS 1.3089	SrpC$_{p}$ (FliS$_{p}$)	I872_RS06615-(I872_RS10965)[b,d]	1753[b]	93	46	123	637	100 + (691)	4<br>21<br>4	SASTSMSNSVSASISASV<br>SQSVSASASASVSVSA<br>SQSVSTSQSAASLSA	3<br>2<br>2

									6	STSASVSA	4
Strep. thoraltensis DSM 12221	SRRP$_{12221p}$	A3IA_RS11895-(A3IA_RS12265)[b,d]	2113[b]	90	49	52	418	83 + (1421)	58<br>14<br>5	SLSTSVSVSESTSTSVSASD<br>SLSTSVSVSE<br>SESVSTSTAK	8<br>4<br>1
Strep. sp. DD12	SRRP$_{DD12}$	STRDD12_RS03215	1848	94	26	93	534	1067	95	SVSQSESAST	30
Strep. sp. DD12	SRRP$_{DD12\text{-}1p}$	STRDD12_RS02690[d]	4608	85	29	84	2352[g]	2002	192	SLSTSLSTSE	116
**Pathogens/Clinical**											
L. gasseri 987_LJOH[k]	SRRP$_{987p}$	(ADF22_RS09755)-ADF22_RS09750[b]	2232[b]	(61)	(3)	(154)	(704) + 681[g]	561	45	SLSNSLSQSE	35
Strep. parasanguinis FW213	Fap1	*fap1*	2587	85	34	72	330	2027	167	SESVSESVSESV	56
Strep. parasanguinis ATCC 903	SRRP$_{903}$[i]	HMPREF8577_RS10255	2064	91	10	94	536	451	21	SQSASLSTSASESASVSA	4
Strep. parasanguinis 318_SPAR	FliS	ADH26_RS04780	2377	87	30	71	586	1568	91<br>12	SVSTSESVSTSE<br>SQSVSQSQSAST	20<br>1
Strep. parasanguinis 540.rep2_SPAR	FliS	ADJ63_RS09645	2333	87	30	71	586	1524	94<br>13	SVSTSESVSTSE<br>SQSVSQSQSAST	16<br>1
Strep. parasanguinis MGH413	GspB$_{MGH413}$	TZ97_01583	1439	87	30	58	390	839	52	SVSTSESVSTSE	10
Strep. gordonii M99	GspB	*gspB*	3072	90	32	114	367	2425	196	SASTSASVSASE	137
Strep. gordonii DL1	Has	*hsa*	2178	90	32	105	224	1683	131	SASTSASVSASE	81
Strep. sanguinis SK36	SrpA	SSA_0829	1625	91	27	125	203	1134	85	SASTSASVSAST	67
Strep. sanguinis SK678	SRRP$_{SK678}$	HMPREF9392_0959	1782	91	27	125	216	1279	97	SASTSASVSAST	77
Strep. sanguinis SK1058	SRRP$_{SK1058}$	HMPREF9395_1527	2843	91	27	125	414	2142	166	SASTSASVSAST	136
Strep. sanguinis SK115	SRRP$_{SK115}$	HMPREF9382_0971	2848	91	27	125	367	2194	172	SASTSASVSAST	143
Staph. aureus COL	SraP	SACOL2676	2261	90	27	112	524	1450	50	STSTSLSDST	3
Staph. aureus N315	SraP	SA2447	2271	90	27	112	524	1460	50	STSTSLSDST	3
Staph. haemolyticus JCSC1435	SraP$_{JCSC1435}$	SH0326	3608	90	37	174	196	3058	155<br>7	SVSDSTSASTSLSASTST<br>STANSQSASTSTST	30<br>4

Strep. agalactiae NEM316	Srr-1	gbs1529	1310	92	35	72	442	632	51	SASTSASTSAST	19
Strep. agalactiae J48	Srr-2	*srr-2*	1115	90	32	70	339	544	40	SESVSSSESVSS	9
Strep. pneumoniae ATCC 700669	PsrP	*psrP*	4433	80	0	42	252	4019	324	SASTSASTSAST	116
Strep. salivarius FDAARGOS_259[l]	SrpA[i]	A6J87_08425	2345	90	21	104	572	499	8<br>6<br>20	SQSVSQSESEVVSK<br>SQSVSESEVVSK<br>SASASESASASE	7<br>4<br>10
Strep. salivarius FDAARGOS_259[l]	SrpB	A6J87_08430	1974	90	21	103	273	1436	47<br>2<br>27	SQSESNSESASESASVSE<br>SnSSSLSnSnnnSn<br>SASASASVSASE	12<br>-<br>4
Strep. salivarius FDAARGOS_259[l]	SrpC	A6J87_08435	2524	90	32	132	610	1613	62<br>6<br>3<br>4<br>9	SQSVSNSESASESESTSE<br>SQSQSASTSASLSVVKSE<br>SISSSLSLSAQQSVSK<br>SVSSSLSESLKTSV<br>SnSASnSASVSASA	4<br>2<br>2<br>4<br>1
Strep. salivarius CCHSS3[l]	SrpA$_{SS3}$	SALIVB_0638	1839	90	36	84	486	1094	63	SASESASTSASMSAST	44
Strep. salivarius CCHSS3[l]	SrpB$_{SS3p}$	SALIVB_0639[d]	4591	90	36	84	1479[g]	2836	174	SASESASTSASMSAST	121
Strep. salivarius CCHSS3[l]	SrpC$_{SS3}$	SALIVB_0640	3146	90	36	76	823[g]	2072	121	SASESASTSASMSAST	85
Strep. suis ISU2912	SRRP$_{ISU2912}$	XK27_04470	3394	90	36	107	2094[g]	1015	81	SASTSASTSAST	63
Strep. suis LSS99	GspB$_{LSS99}$	ERS132461_00137	3356	90	36	107	1901[g]	1171	94	SASTSASTSAST	79
Strep. suis LSS38	GspB$_{LSS38}$	ERS132400_01130	2603	90	36	107	1931[g]	387	28	SASTSASTSAST	22
Strep. suis LSS88	GspB$_{LSS88}$	ERS132450_00689	2497	[90][m]	36	103	1459[g]	754	59	SASTSASTSAST	50
Strep. suis LSS32	GspB$_{LSS32}$	ERS132394_00457	2273	90	36	123	1263[g]	717	54	SASTSASTSAST	11
Strep. suis CZ130302	SssP1$_{p}$[n]	CVO91_04815	4647	90	34	72	2234[g]	1378	113<br>26	SASTSASTSAST<br>SASTSASTSAST[o]	111<br>24
Strep. suis YS54	SRRP$_{YS54p}$	SST61_RS0109470[d]	2420	90	36	107	2105[g]	82	6	SASTSASTSAST	4
Strep. suis R61	SRRP$_{R61p}$	SSUR61_RS00005[d]	1651	[90][m]	39	112	1268[p]	98	8	SASTSASTSAST	6
Strep. suis 92-4172[q]	Fap1$_{92\text{-}4172}$	SST27_RS0104530	2510	85	25	146	556[r]	1651	137	SESVSESVSESV	37
Strep. sp. 449_SSPC	SRRP$_{449}$	ADH51_RS02350	1491	90	32	92	273	951	18<br>30	SQSVSNSESASESASVSE<br>SASTSASVSASE	5<br>6

[a] Subscript p after the name of a SRRP indicates encoding by a probable pseudogene

[b] Estimated sizes of pseudo-SRRP and domains reconstructed from adjacent pseudogene-encoded fragments if reading frame had not been disrupted. Locus tags and values in round brackets are those of adjacent pseudogene fragments and encoded residues. In the majority of cases, the BR domain was encoded by an uninterrupted reading frame

[c] Originally the C-terminal border of the $SRRP_{100\text{-}23}$ BR domain was predicted to extend further and this longer BR domain was the one expressed in *E. coli*

[d] Genes encoding all the features of a SRRP, except for a C-terminal LPXTG cell wall anchor sometimes with a truncated SRR-2 domain, that are therefore capable of producing a C-terminally truncated SRRP or, alternatively, could be considered to be pseudogenes

[e] Possible intact SRRP gene with partial sequence at the end of a draft genome contig

[f] Gene incorrectly annotated in genome; full-length SRRP translated in one reading frame, except for the case of *Strep. salivarius* ATCC 25975 $SrpB_p$ which lacks a complete LPKTG cell wall anchor (LPKFI)

[g] The majority of BR domains are 200-600 aa in size. Exceptions are the three *L. johnsonii* SRRPs that are annotated as haemagglutinins ($SRRP_{N6.2}$, $SRRP_{533\text{-}2}$ and $SRRP_{6026p}$); the six *Strep. salivarius* SRRPs $SrpB_{25975p}$, $SrpC_{25975p}$, $SrpB_{57.Ip}$, $SrpC_{57.Ip}$, $SrpB_{SS3p}$ and $SrpC_{SS3}$, whose BR domains contain an L-type lectin region, a single Rib/alpha/Esp surface antigen repeat and a hyperosmolarity resistance protein Ebh (N-terminal domain) region; $SRRP_{DD12\text{-}1p}$ whose BR contains the Rib/alpha/Esp and Ebh (N-terminal domain) regions but lacks the L-type lectin region; and the seven *Strep. suis* SRRPs whose BRs contain complete or partial bacterial group 3 immunoglobulin-like domains and in two cases ($GspB_{LSS32}$ and $SRRP_{YS54p}$), also contain a FlgD Tudor-like domain; in addition, $SssP1_p$-BR contains a Herpes virus major outer envelope glycoprotein (BLLF1) domain

[h] Translated in the opposite reading frame to that annotated in the genome from nt 112572–115183

[i] These SRRPs also contain varying numbers of MucBP repeat motifs upstream or downstream of the SRR-2 domain or in some cases flanking both sides of the SRR-2 (see Figure S1)

[j] Formerly annotated as full-length SrpA ORF9R in GenBank accession number U96166 (locus SCU96166)

[k] A clinical isolate from the wound of an intensive care unit patient, originally identified as *L. johnsonii*

[l] Although members of the viridans group streptococci are classed mainly as commensal, probiotic or lantibiotic strains, some can cause major infections and strains FDAARGOS_259 and CCHSS3 are clinical isolates of *Strep. salivarius* from human blood

[m] Atypical SecA2-SecY2 signal sequences that may not be functional

[n] Although $SssP1_p$ lacks an LPXTG cell wall anchor, it contains additional N3 and SRR-3 domains at its C-terminus which may be involved in tethering the protein to the bacterial cell surface

[o] The SRR consensus sequence of the additional SRR-3 domain of $SssP1_p$ is shown separately to that of the SRR-2 domain

[p] Unlike the other *Strep. suis* SRRPs, this BR domain does not contain a group 3 bacterial immunoglobulin-like domain

[q] This strain also contains a pseudo-Fap1 (SST27_RS0108845) of 1859 aa that lacks a secretion signal, AST, SRR-1 and part of the BR domains, and a pseudo-SRRP (SST27_RS0103965-_RS103960) of about 938 aa

[r] This BR domain also contains a group 3 bacterial immunoglobulin-like domain
