# Supplementary material for: Serine-rich repeat proteins from gut microbes
Source: Gut Microbes. 2019 Apr 29;11(1):102–17. doi: 10.1080/19490976.2019.1602428 (PMC6973325; doi:10.1080/19490976.2019.1602428)
Supplement: Supplemental Material [file kgmi-11-01-1602428-s001.zip › Supplementary information/new_Table S4.pdf]

Table S4. The isoelectric point (pI) of each BR domain, calculated from the aa sequence using ExPASy ProtParam, is colour-coded as follows for the arbitrarily set pI limits: red, "strong acidic" (pH <5.55); yellow, "weak acidic" (pH 5.55-7.00); blue, basic (pH >8.00).

		SRRP-BR	pI
Commensals/Food sources	1	L.reuteri_ATCC_53608_SRRP	4.80
	2	L.reuteri_lp167-67_SRRP	4.72
	3	L.reuteri_I5007_pseudo-SRRP-1	4.81
	4	L.reuteri_121_SRRP	4.80
	5	L.reuteri_ZLR003_SRRP	4.76
	6	L.reuteri_pg-3b_SRRP	4.80
	7	L.reuteri_KLR1001_SRRP	4.91
	8	L.reuteri_KLR1002_SRRP	4.75
	9	L.reuteri_KLR3004_SRRP	4.67
	10	L.reuteri_20-2_SRRP	4.60
	11	L.reuteri_3c6_SRRP	4.60
	12	L.reuteri_lpuph_pseudo-SRRP-1	4.52
	13	L.reuteri_LR0_pseudo-SRRP-1	4.52
	14	L.reuteri_TD1_pseudo-SRRP	4.52
	15	L.reuteri_LTH5448_pseudo-SRRP	4.39
	16	L.reuteri_KLR4001_SRRP	4.59
	17	L.reuteri_100-23_SRRP	4.81
	18	L.reuteri_480_44_pseudo-SRRP	4.40
	19	L.reuteri_482_46_pseudo-SRRP	4.40
	20	L.reuteri_482_54_pseudo-SRRP	4.00
	21	L.reuteri_484_39_pseudo-SRRP	4.51
	22	L.reuteri_lpuph_pseudo-SRRP-2	4.40
	23	L.reuteri_LR0_pseudo-SRRP-2	4.40
	24	L.reuteri_I49_SRRP	4.59
	25	L.reuteri_TD1_SRRP	4.40
	26	L.reuteri_LTH5448_SRRP	4.57
	27	L.reuteri_LTH2584_RS06575_pseudo-SRRP-1	4.47
	28	L.reuteri_TMW1.656_00083_pseudo-SRRP-1	4.47
	29	L.reuteri_100-23_pseudo-SRRP	4.40
	30	L.reuteri_1366_SRRP	4.89
	31	L.reuteri_KLR2001_pseudo-SRRP-2	5.24
	32	L.reuteri_KLR3005_pseudo-SRRP-2	5.05
	33	L.reuteri_pg-3b_pseudo-SRRP	5.05
	34	L.reuteri_TMW1.112_RS02820_pseudo-SRRP	4.37
	35	L.reuteri_LTH2584_RS10630_pseudo-SRRP-2	5.51
	36	L.reuteri_TMW1.656_00668_pseudo-SRRP-2	5.23
	37	L.reuteri_ATCC_53608_pseudo-SRRP	5.22
	38	L.reuteri_3c6_pseudo-SRRP	5.30
	39	L.reuteri_lp167-67_pseudo-SRRP	5.01
	40	L.reuteri_I5007_pseudo-SRRP-2	5.22
	41	L.reuteri_121_pseudo-SRRP	5.22
	42	L.reuteri_ZLR003_pseudo-SRRP	5.22
	43	L.reuteri_KLR1001_pseudo-SRRP	5.22
	44	L.reuteri_KLR1002_pseudo-SRRP	5.22
	45	L.reuteri_KLR1004_SRRP	5.22
	46	L.reuteri_KLR2002_pseudo-SRRP-2	5.22
	47	L.reuteri_KLR2003_pseudo-SRRP-2	5.22
	48	L.reuteri_KLR2004_pseudo-SRRP-2	5.22
	49	L.reuteri_KLR2007_pseudo-SRRP-2	5.22
	50	L.reuteri_KLR2008_pseudo-SRRP-2	5.22
	51	L.reuteri_KLR3002_pseudo-SRRP-2	5.22
	52	L.reuteri_KLR3003_pseudo-SRRP-2	5.22
	53	L.reuteri_KLR3004_pseudo-SRRP	5.22
	54	L.reuteri_KLR3006_pseudo-SRRP-2	5.22
	55	L.reuteri_CECT8605_pseudo-SRRP-2	5.22
	56	L.mucosae_LM1_pseudo-SRRP	6.10
	57	L.johnsonii_NCC533_LJ1711_SRRP-2	4.53
	58	L.johnsonii_DPC6026_pseudo-SRRP-2	4.55
	59	L.johnsonii_N6.2_SRRP	4.64
	60	L.johnsonii_NCC533_LJ0391_SRRP-1	5.11
	61	L.johnsonii_N6.2_pseudo-SRRP	4.85
	62	L.johnsonii_16_pseudo-SRRP	4.90
	63	L.johnsonii_W1_pseudo-SRRP	4.53
	64	L.johnsonii_DPC_6026_pseudo-SRRP-1	5.15
	65	L.oris_F0423_SraP	5.09
	66	L.oris_PB013-T2-3_pseudo-SraP	5.09
	67	L.oris_F0423_SRRP	5.11
	68	L.oris_PB013-T2-3_SRRP	5.10
	69	L.fructivorans_DmCS_002_SRRP	4.53
	70	L.salivarius_NIAS840_SRRP	5.50
	71	L.salivarius_NIAS840_pseudo-SRRP	6.03
	72	L.salivarius_JCM1046_SRRP	4.76
	73	L.salivarius_SMXD51_pseudo-SRRP-1	5.27
	74	L.salivarius_SMXD51_pseudo-SRRP-2	5.82
	75	L.nagelii_DSM_13675_pseudo-SRRP	4.11
	76	Lc.lactis_subsp._cremoris_KW2_SRRP	4.24
	77	Strep.sp._DD12_00537_pseudo-SRRP-1	5.07
	78	Strep.sp._DD12_00645_SRRP	5.79
	79	Strep.thoraltensis_DSM_12221_pseudo-SRRP	4.70
	80	Strep.mitis_B6_MonX	6.86
	81	Strep.oralis_Uo5_MonX	5.33
	82	Strep.cristatus_CC5A_SrpA	6.24
	83	Strep.cristatus_ATCC_51100_SraP	6.64
	84	Strep.cristatus_ATCC_51100_SrpC	8.29
	85	Strep.cristatus_AS_1.3089_pseudo-SrpC	6.34
	86	Strep.vestibularis_F0396_FliS	6.45
	87	Strep.salivarius_JIM8777_SrpC	5.56
	88	Strep.salivarius_ATCC_27945_pseudo-SrpC	6.32
	89	Strep.salivarius_JF_SrpC	6.98
	90	Strep.salivarius_NCTC_8618_SrpC	6.32
	91	Strep.salivarius_HSISS4_SrpC	5.97
	92	Strep.salivarius_K12_SrpC	5.74
	93	Strep.salivarius_JIM8777_SrpB	4.78
	94	Strep.salivarius_ATCC_27945_SrpB	4.90
	95	Strep.salivarius_HSISS4_SrpB	4.92
	96	Strep.salivarius_JF_SrpB	5.06
	97	Strep.salivarius_NCTC_8618_SrpB	5.06
	98	Strep.salivarius_JIM8777_SrpA	4.88
	99	Strep.salivarius_ATCC_27945_SrpA	4.83
	100	Strep.salivarius_JF_SrpA	4.88
	101	Strep.salivarius_NCTC_8618_SrpA	4.88
	102	Strep.salivarius_HSISS4_pseudo-SrpA	5.07
	103	Strep.salivarius_ATCC_25975_SrpA	4.59
	104	Strep.salivarius_57.I_pseudo-SrpA	4.52
	105	Strep.salivarius_ATCC_25975_pseudo-SrpB	5.74
	106	Strep.salivarius_57.I_pseudo-SrpB	5.56
	107	Strep.salivarius_ATCC_25975_pseudo-SrpC	5.18
	108	Strep.salivarius_57.I_pseudo-SrpC	5.16
Pathogens/Clinical isolates	109	Strep.salivarius_CCHSS3_SrpC	5.17
	110	Strep.salivarius_FDAARGOS_259_SrpA	4.85
	111	Strep.salivarius_CCHSS3_SrpA	4.48
	112	Strep.salivarius_FDAARGOS_259_SrpB	5.04
	113	Strep.salivarius_CCHSS3_pseudo-SrpB	5.58
	114	Strep.salivarius_FDAARGOS_259_SrpC	6.45
	115	Strep.parasanguinis_318_SPAR_FliS	5.87
	116	Strep.parasanguinis_540.rep2_SPAR_FliS	5.87
	117	Strep.parasanguinis_ATCC_903_SRRP	4.95
	118	Strep.sp._449_SSPC_SRRP	5.06
	119	Strep.gordonii_DL1_Hsa	9.03
	120	Strep.gordonii_M99_GspB	9.17
	121	Staph.haemolyticus_JCSC1435_SraP	9.25
	122	Strep.pneumoniae_ATCC_700669_PsrP	9.66
	123	Strep.parasanguinis_FW213_Fap1	5.35
	124	Strep.parasanguinis_MGH413_GspB	5.05
	125	Strep.suis_YS54_pseudo-SRRP	4.95
	126	Strep.suis_ISU2912_SRRP	5.01
	127	Strep.suis_LSS99_GspB	5.32
	128	Strep.suis_LSS38_GspB	4.94
	129	Strep.suis_LSS88_GspB	4.99
	130	Strep.suis_LSS32_GspB	5.11
	131	Strep.suis_R61_pseudo-SRRP	4.76
	132	Strep.suis_92-4172_Fap1	4.61
	133	Staph.aureus_N315_SraP	5.69
	134	Strep.sanguinis_SK36_SrpA	5.94
	135	Strep.sanguinis_SK678_SRRP	6.61
	136	Strep.sanguinis_SK115_SRRP	8.72
	137	Strep.sanguinis_SK1058_SRRP	5.24
	138	Strep.agalactiae_NEM316_Srr-1	4.78
	139	Strep.agalactiae_J48_Srr-2	4.67
	140	L.gasseri_987_LJOH_pseudo-SRRP	4.86
